# Cell-type-specific autophagy in root-hair-forming cells is essential for salt stress tolerance in *Arabidopsis thaliana*

**DOI:** 10.1038/s41477-026-02285-w

**Published:** 2026-05-06

**Authors:** Jierui Zhao, Peng Gao, Sunhuan Xiang, Christian Löfke, Kai Ching Yeung, Yixuan Chen, Liwen Jiang, Yasin Dagdas

**Affiliations:** 1 Gregor Mendel Institute of Molecular Plant Biology, Austrian Academy of Sciences, Vienna BioCenter, Vienna, Austria; 2 Vienna BioCenter PhD Program, Doctoral School of the University at Vienna and Medical University of Vienna, Vienna, Austria; 3 School of Life Sciences, State Key Laboratory of Agrobiotechnology, Chinese University of Hong Kong, Hong Kong, China; 4 AoE Centre for Organelle Biogenesis and Function, AoE Centre for Plant Vacuole Biology and Biotechnology, Centre for Cell and Developmental Biology, Chinese University of Hong Kong, Hong Kong, China; 5 Institute of Plant Molecular Biology and Agricultural Biotechnology, Chinese University of Hong Kong, Hong Kong, China; 6 CUHK Shenzhen Research Institute, Shenzhen, China; 7 Centre for Organismal Studies, Heidelberg University, Heidelberg, Germany; 8 Cluster of Excellence GreenRobust, Heidelberg University, Heidelberg, Germany

**Keywords:** Plant cell biology, Cell biology

## Abstract

Autophagy is a vital cellular quality control pathway that maintains cellular homoeostasis under changing environments. While the organismal phenotypes of autophagy-deficient plants under stress are well characterized, the contribution of cell-type-specific autophagy responses to whole-plant homoeostasis remains poorly understood. Here we show that root-hair-forming cells (trichoblasts) in *Arabidopsis thaliana* exhibit higher autophagic flux than adjacent non-hair cells (atrichoblasts). This differential autophagy is genetically linked to cell fate determination during early development. Mutants with disrupted trichoblast or atrichoblast identity lose the autophagy distinction between these cell types. Functional analyses reveal that elevated autophagy in trichoblasts is essential for sodium ion sequestration in vacuoles—a key mechanism for salt stress tolerance. Disrupting autophagy specifically in trichoblasts impairs sodium accumulation and reduces plant survival under salt stress. Conversely, cell-type-specific complementation restores both sodium sequestration and stress tolerance. Our findings uncover a cell-type-specific autophagy program in root hairs and demonstrate how developmental cues shape autophagy to enhance stress resilience. This work establishes a direct link between cell identity, autophagy and environmental adaptation in plants.

## Abstract (web summary)

This study reveals that enhanced autophagy in root-hair-forming cells helps *Arabidopsis* store excess sodium, control oxidative stress and tolerate salt stress.

## Main

Autophagy, a conserved eukaryotic degradation pathway, plays a pivotal role in maintaining cellular homoeostasis by recycling damaged organelles, protein aggregates and microorganisms^1^. It is orchestrated by a suite of autophagy-related (ATG) proteins that mediate the de novo formation of a double-membraned vesicle, termed the autophagosome, which captures and delivers the cargo to the vacuole for breakdown^2,3^. Autophagosome biogenesis is initiated by the ATG1 kinase complex (comprising ATG1, ATG13, ATG11 and ATG101), which integrates stress signals from upstream regulators such as TOR and SnRK1 kinase complexes to initiate autophagosome biogenesis^3,4^. Phagophore expansion depends on the phosphatidylinositol 3-kinase complex (VPS34, ATG6 and VPS15), and membrane transfer is facilitated by proteins such as ATG9 and ATG2 (refs. ^1,3^). Autophagosome maturation requires the ubiquitin-like conjugation systems (ATG3, ATG4, ATG7 and ATG12–ATG5–ATG16) responsible for ATG8 lipidation—a hallmark of autophagosome formation^1,5^. While ATG proteins orchestrate autophagosome biogenesis, cargo selection and recruitment are mediated by selective autophagy receptors, such as NBR1 and ATI1 (refs. ^6–9^). Selective autophagy receptors on the one hand interact with ATG8 via conserved ATG-interacting motifs and on the other hand contain cargo recognition domains for selective cargo recruitment^1,10,11^.

Plant autophagy is a critical adaptive mechanism enabling survival during environmental stress^12,13^. Under abiotic stress, such as nutrient deprivation, drought and salinity, autophagy reallocates cellular resources to sustain metabolic functions and delay senescence^13,14^. The functional importance of autophagy in plants is underscored by the phenotypic consequences observed in autophagy-deficient mutants (*atg* mutants), which exhibit abnormal organ development, premature senescence, hypersensitivity to biotic and abiotic stresses, and metabolic imbalances^15–17^. While organismal outcomes of autophagy defects are well established, emerging evidence emphasizes its cell-type-specific functions, allowing tailored responses across plant tissues^18^. For example, in the *Arabidopsis* root cap, autophagy facilitates the programmed death and clearance of root cap border cells, a process critical for organized cell separation and root growth^18,19^. The disruption of ATG5 in the root cap via tissue-specific CRISPR mutagenesis impairs vacuolization and the removal of dying cells, demonstrating the developmental precision of autophagy in a spatially restricted context^18^. Similarly, cell-type-specific autophagy has been shown to play important roles in leaf abscission^20,21^. However, the degree to which cell-type-specific autophagy responses contribute to stress tolerance remains largely unknown.

*Arabidopsis* roots offer an ideal system to investigate cell-type-specific autophagy, as the patterning of cell types that have very different metabolic and homeostatic demands is well established. In particular, the trichoblasts and atrichoblasts, which are adjacent to each other and differentiate into root hair and non-root-hair cells^22^, provide an excellent system to study cell-type-specific homeostatic responses. The differentiation of trichoblasts (root hair cells) and atrichoblasts (non-hair cells) in the *Arabidopsis* root epidermis is a complex process regulated by a combination of positional signalling, transcriptional networks and hormonal cues^23,24^. The fate of *Arabidopsis* root epidermal cells is determined by their position relative to the underlying cortical cells. Cells in contact with two cortical cells (T position) become trichoblasts, while those in contact with only one cortical cell (A position) become atrichoblasts^23,24^. The differentiation of trichoblasts and atrichoblasts is regulated by a network of transcription factors. In A-positioned cells, the MYB transcription factor WEREWOLF (WER) forms a complex with the bHLH proteins GLABRA3 or ENHANCER OF GLABRA3 and the WD40 protein TRANSPARENT TESTA GLABRA1 (refs. ^23,24^). This complex promotes the expression of the homeodomain protein GLABRA2 (GL2), which inhibits the expression of downstream genes related to root hair formation, leading to the atrichoblast fate^23,24^. In T-positioned cells, the R3 MYB protein CAPRICE (CPC) (or its paralogues TRYPTYCHON (TRY) and ENHANCER OF TRY AND CPC1) replaces WER in the complex, preventing GL2 expression and allowing the cells to adopt the trichoblast fate^23,24^. A previous study reported autophagic flux differences between root hair cells and non-root-hair cells^25^. However, the physiological and genetic basis underlying this difference remained unknown. Understanding how autophagy links with the cell-type-specific transcriptional programs in *Arabidopsis* roots may reveal novel regulatory hubs that mediate stress adaptation and developmental plasticity.

Here we dissected autophagic flux patterns in trichoblasts and atrichoblasts during salt stress. Using a large suite of reporters, we show that trichoblast cells have higher autophagic flux than the adjacent atrichoblast cells. This is encoded by the trichoblast developmental program, as autophagic flux differences disappeared in genetic mutants that change cell fate. Cell-type-specific CRISPR mutagenesis and complementation experiments revealed that higher autophagic flux in trichoblasts is crucial for salt stress tolerance. Altogether, by mapping autophagy dynamics at cellular resolution, we have uncovered a cell-type-specific autophagy response that is crucial for salt stress resilience.

## Results

### Trichoblasts exhibit higher autophagic flux in the *Arabidopsis* root maturation zone

**Fig. 1:**
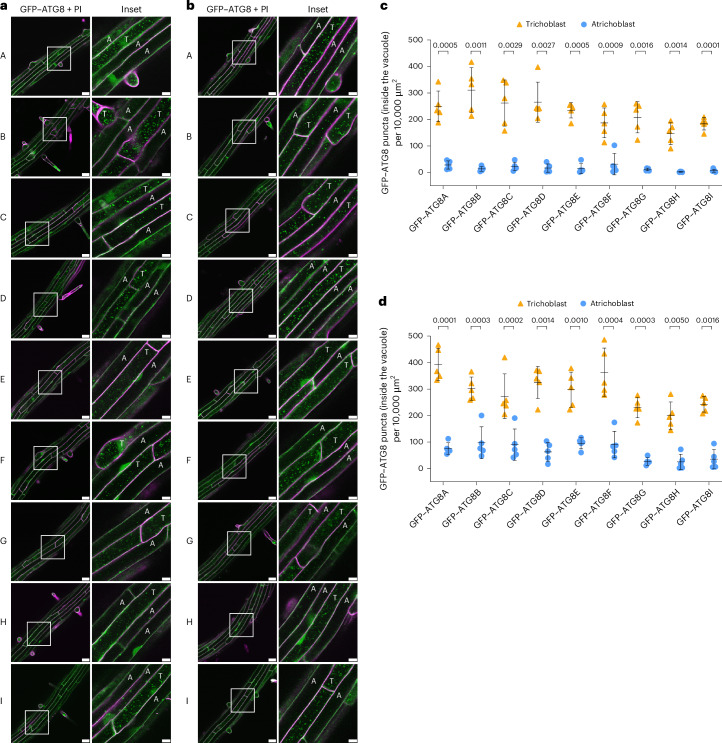
Trichoblasts exhibit significantly higher autophagic flux than the adjacent atrichoblast cells under both control and NaCl treatments in the *A. thaliana* root maturation zone. **a**,**b**, Confocal microscopy images of the trichoblasts and atrichoblasts in the root maturation zone of *Arabidopsis* wild-type Col-0 expressing *ProUBQ10*::*GFP-ATG8A–I* isoforms under control (**a**) and NaCl (**b**) treatment. Five-day-old *Arabidopsis* seedlings were incubated in control 1/2 MS media containing 2 μM conA for 2 h (**a**) or 1/2 MS media containing 50 mM NaCl + 1 μM conA for 40–60 min (**b**) before imaging. Representative images of five replicates are shown. The areas highlighted by white boxes in the GFP–ATG8 + PI panels are further enlarged and presented in the inset panels. Scale bars, 30 μm. Inset scale bars, 10 μm. Green indicates GFP–ATG8A–I isoforms; magenta indicates propidium iodide (PI) dye. T, trichoblast; A, atrichoblast. **c**,**d**, Quantification of GFP–ATG8 puncta inside the vacuole per normalized area (10,000 μm^2^) of the trichoblasts and atrichoblasts imaged in **a** (**c**) or **b** (**d**). The bars indicate the mean ± s.d. of five replicates. Two-tailed and paired Student *t*-tests were performed to analyse the significance of GFP–ATG8 puncta density differences between the trichoblasts and the atrichoblasts. Exact *P* values are centred directly above the comparison brackets.

**Extended Data Fig. 1:**
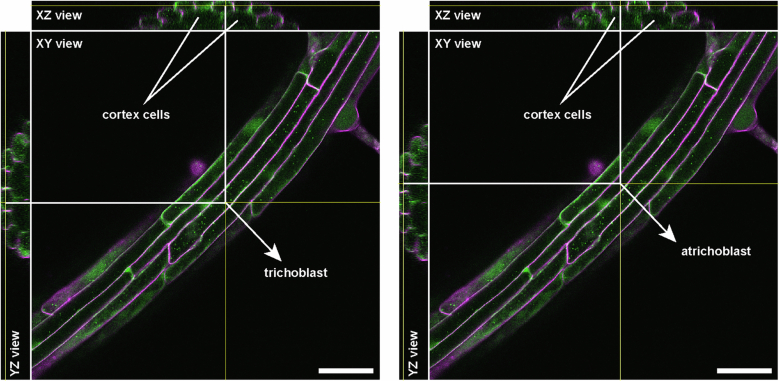
Schematic confocal images illustrating the approach used to distinguish trichoblasts from atrichoblasts through confocal microscopy. To identify trichoblast and atrichoblast cells, we analyzed XZ and YZ views of Z-stack images. Trichoblast (root hair forming) cells are identified as cells that are adjacent to two cortex cells and atrichoblast (non-root hair forming) as cells that are adjacent to one cortex cell. Green color, GFP-ATG8A. Magenta color, propidium iodide dye (for cell wall staining). Scale bars, 50 μm. Yellow lines show the alignment of XY, XZ and YZ views of the target cell. Representative images of 5 replicates are shown.

**Extended Data Fig. 2:**
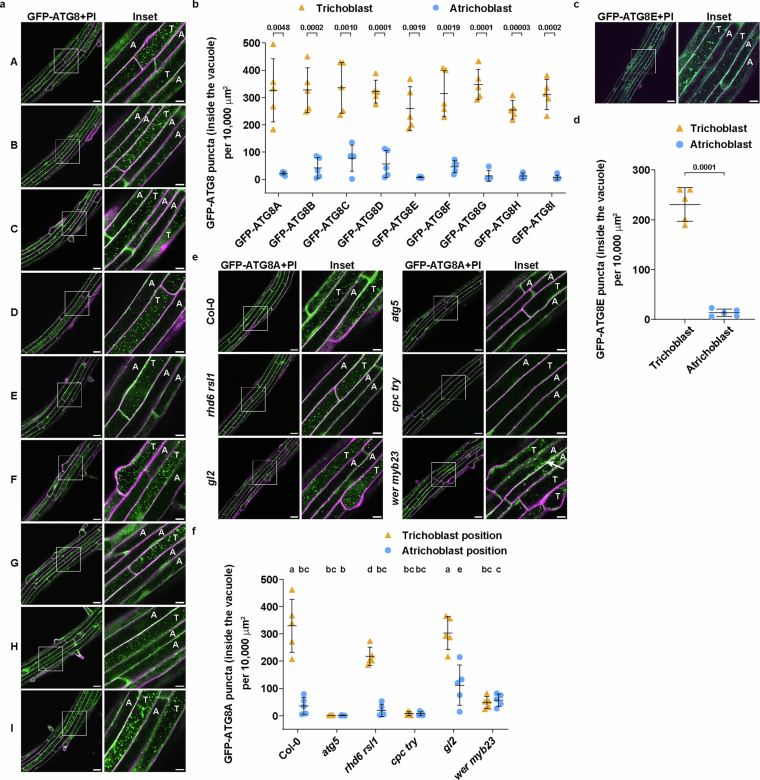
Measurement of autophagic flux in the trichoblasts and atrichoblasts of Arabidopsis root maturation zone. **a**. Confocal microscopy images of the trichoblasts and atrichoblasts in the root maturation zone of Arabidopsis wildtype Col-0 expressing ProUBQ10:GFP-ATG8A–I isoforms under nitrogen-starvation conditions. 5 days old Arabidopsis seedlings were incubated in nitrogen-deficient 1/2 MS media containing 2 μM concanamycin A for 2 h before imaging. Representative images of 5 replicates are shown. Area highlighted in the white-boxed region in the GFP-ATG8 + PI panel was further enlarged and presented in the inset panel. Scale bars, 30 μm. Inset scale bars, 10 μm. Green color, GFP-ATG8A–I isoforms. Magenta color, propidium iodide dye. T, trichoblasts. A, atrichoblasts. **b**. Quantification of the GFP-ATG8 puncta inside the vacuole per normalized area (10,000 μm^2^) of the trichoblasts and atrichoblasts imaged in **a**. Bars indicate the mean ± SD of 5 replicates. Two-tailed and paired student t tests were performed to analyze the significance of GFP-ATG8 puncta density differences between the trichoblasts and the atrichoblasts. Exact p values are centered directly above the comparison brackets. **c**. Confocal microscopy images of trichoblasts and atrichoblasts of Arabidopsis wildtype Col-0 expressing ProATG8E:GFP-ATG8E. 5 days old Arabidopsis seedlings were incubated in 1/2 MS media containing 2 μM conA for 3 h before imaging. Representative images of 5 replicates are shown. Area highlighted in the white-boxed region in the GFP-ATG8E + PI panel was further enlarged and presented in the inset panel. Scale bars, 30 μm. Inset scale bars, 10 μm. Green color, GFP-ATG8E. Magenta color, propidium iodide dye. T, trichoblasts. A, atrichoblasts. **d**. Quantification of the GFP-ATG8E puncta inside the vacuole per normalized area (10,000 μm^2^) of the trichoblasts and atrichoblasts imaged in **c**. Bars indicate the mean ± SD of 5 replicates. Two-tailed and paired student t test was performed to analyze the significance of GFP-ATG8E puncta density difference between the trichoblasts and the atrichoblasts. Exact p values are centered directly above the comparison brackets. **e**. Confocal microscopy images of epidermal cells in the root maturation zone of Col-0, *atg5*, *rhd6 rsl1*, *cpc* *try*, *gl2* and *wer myb23* expressing ProUBQ10:GFP-ATG8A under nitrogen-starvation conditions. 5-d-old Arabidopsis seedlings were incubated in nitrogen-deficient 1/2 MS media containing 2 μM concanamycin A for 2 h before imaging. Representative images of 5 replicates are shown. Area highlighted in the white-boxed region in the GFP-ATG8A + PI panel was further enlarged and presented in the inset panel. Scale bars, 30 μm. Inset scale bars, 10 μm. Green color, GFP-ATG8A. Magenta color, propidium iodide dye. A, the atrichoblast positions (adjacent to only one cortex cell). Note that the spindle-shaped ER bodies in *wer myb23* (white arrow) were not inside the vacuole and were not counted as GFP-ATG8A puncta for autophagic flux quantification. **f**. Quantification of the GFP-ATG8A puncta inside the vacuole per normalized area (10,000 μm^2^) of the cells at the trichoblast positions and the atrichoblast positions imaged in **e**. Bars indicate the mean ± SD of 5 replicates. Paired repeated measures one-way ANOVA and Fisher’s LSD tests were used to analyze the differences of the number of the GFP-ATG8A puncta between each group. Groups sharing the same letters (a/b/c) are not significantly different, while those with different letters are significantly different. Family-wise significance and confidence level, 0.05 (95% confidence interval).

Trichoblasts and atrichoblasts are organized in a highly structured adjacent manner. To investigate whether these two cell types exhibit distinct autophagic activities, we first measured the autophagic flux in trichoblasts and atrichoblasts in the root maturation zone of *Arabidopsis* wild-type Col-0 expressing *ProUBQ*::*GFP-ATG8A*, under control and two autophagy-inducing conditions (NaCl stress and nitrogen starvation), using confocal microscopy. To accurately identify trichoblasts and atrichoblasts in our live-cell imaging experiments, we adhered to the following criteria: an epidermal cell was classified as a trichoblast if it was adjacent to two cortical cells, whereas it was classified as an atrichoblast if it was adjacent to only one cortical cell (Extended Data Fig. 1). Under both control and autophagy-inducing conditions, confocal microscopy revealed that trichoblasts exhibit significantly higher autophagic flux than the adjacent atrichoblast cells (Fig. 1 and Extended Data Fig. 2a,b). Similar to ATG8A, Col-0 plants expressing the other eight *ProUBQ*-driven ATG8 isoforms (*ProUBQ*::*ATG8B–I*) also exhibited higher autophagic flux in trichoblast cells than in atrichoblasts in the root maturation zone under control, NaCl stress and nitrogen-starvation conditions (Fig. 1 and Extended Data Fig. 2a,b). Wild-type plants expressing native-promoter-driven ATG8E (*ProATG8E*::*GFP-ATG8E*) also had higher flux in trichoblast cells (Extended Data Fig. 2c,d). Collectively, these results demonstrate that trichoblasts in the root maturation zone exhibit significantly higher autophagic activity than the adjacent atrichoblast cells. This observation prompted us to further explore this cell-type-specific autophagic response in more detail.

### Genetic basis of higher autophagic flux in trichoblasts

**Fig. 2:**
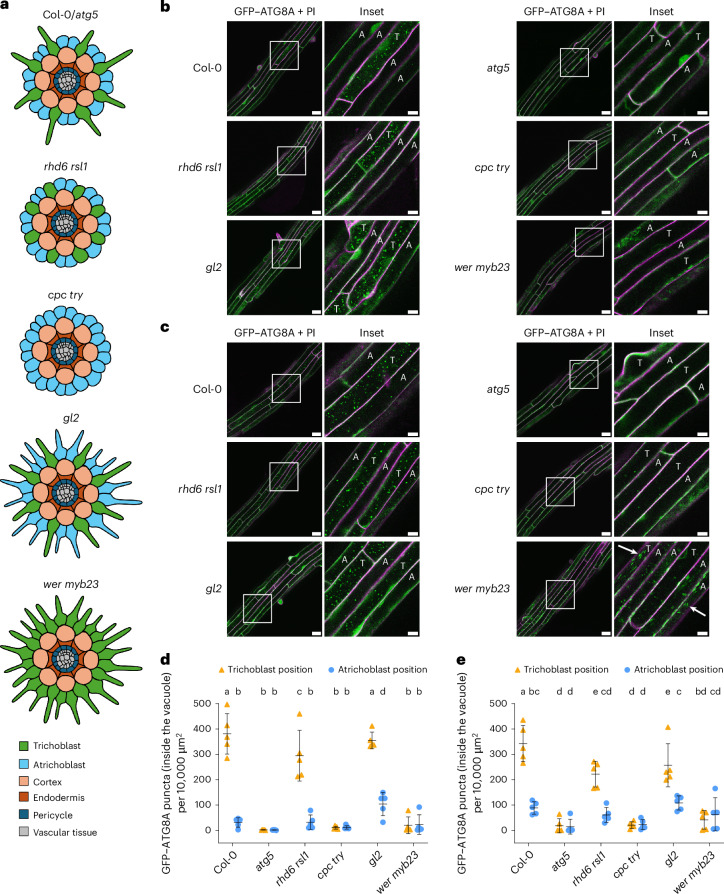
Genetic basis of the autophagic flux difference between trichoblasts and atrichoblasts. **a**, Trichoblast–atrichoblast distribution patterns in *Arabidopsis* wild-type Col-0, the autophagy-defective mutant *atg5* and the root-hair-development mutant lines *rhd6* *rsl1*, *cpc* *try*, *gl2* and *wer* *myb23*. **b**,**c**, Confocal microscopy images of epidermal cells in the root maturation zone of Col-0, *atg5*, *rhd6* *rsl1*, *cpc* *try*, *gl2* and *wer* *myb23* expressing *ProUBQ10*::*GFP-ATG8A* under control (**b**) or NaCl (**c**) treatment. Five-day-old *Arabidopsis* seedlings were incubated in either control 1/2 MS media containing 2 μM conA for 2 h (**b**) or 1/2 MS media containing 50 mM NaCl + 1 μM conA for 40–60 min (**c**) before imaging. Representative images of five replicates are shown. The areas highlighted by white boxes in the GFP–ATG8A + PI panels are further enlarged and presented in the inset panels. Scale bars, 30 μm. Inset scale bars, 10 μm. Green indicates GFP–ATG8A; magenta indicates PI dye. T, trichoblast position (adjacent to two cortex cells); A, atrichoblast position (adjacent to only one cortex cell). Note that the spindle-shaped ER bodies in *wer* *myb23* (white arrows in **c**) were not inside the vacuole and were not counted as GFP–ATG8A puncta for autophagic flux quantification. **d**,**e**, Quantification of GFP–ATG8A puncta inside the vacuole per normalized area (10,000 μm^2^) of the cells at the trichoblast positions and the atrichoblast positions imaged in **b** (**d**) or **c** (**e**). The bars indicate the mean ± s.d. of five replicates. Paired repeated-measures one-way analysis of variance (ANOVA) and Fisher’s least significant difference (LSD) tests were used to analyse the differences in the numbers of GFP–ATG8A puncta between each group. Groups sharing the same letters (a/b/c/d/e) are not significantly different, while those with different letters are significantly different. The family-wise significance and confidence level was 0.05 (95% confidence interval).

The development of trichoblasts and atrichoblasts is orchestrated by a well-characterized genetic program^23,24^. While the formation of root hairs from trichoblasts at the maturation zone results in obvious surface differences, the determination of cell type identity occurs at the meristematic zone^26^. Given that both root hair structure and cell type identity could contribute to the observed differences in autophagic activity, we investigated autophagic activity in a series of *Arabidopsis* root epidermal development mutants. We selected four representative mutants that were previously shown to affect cell fate and identity: *rhd6* *rsl1*, *cpc* *try*, *gl2* and *wer* *myb23* (Fig. 2a). *rhd6* *rsl1* mutants have ectopic non-hair cells at trichoblast positions, while *gl2* mutants display ectopic hair cells at atrichoblast positions. However, both *rhd6* *rsl1* and *gl2* mutants retain meristematic cell type determination, as evidenced by their meristematic vacuolar biogenesis phenotype^26^. In contrast, *cpc* *try* and *wer* *myb23* mutants lose cell type identity; *cpc* *try* mutants have ectopic non-hair cells, whereas *wer* *myb23* mutants display ectopic hair cells (Fig. 2a). To test the genetic basis of higher autophagic flux in trichoblast cells, we expressed GFP-ATG8A in these four mutants, as well as in *atg5* as an autophagy-deficient control, and performed live-cell imaging experiments similar to those described in Fig. 1. Flux analysis revealed that *rhd6* *rsl1* and *gl2* mutants maintained the autophagic flux difference, whereas *cpc* *try* and *wer* *myb23* mutants lost this difference. This suggests that cell fate defined at the meristematic zone underlies the differential autophagic activity between trichoblasts and atrichoblasts (Fig. 2b–e and Extended Data Fig. 2e,f). However, the presence of root hair structures also appeared to slightly enhance autophagic flux, as evidenced by the slightly lower autophagic flux in trichoblast-positioned cells of *rhd6* *rsl1* mutants than in Col-0 and the *gl2* mutant, and the slightly higher autophagic flux in atrichoblast-positioned cells of the *gl2* mutant than in the other lines (Fig. 2b–e and Extended Data Fig. 2e,f).

**Fig. 3:**
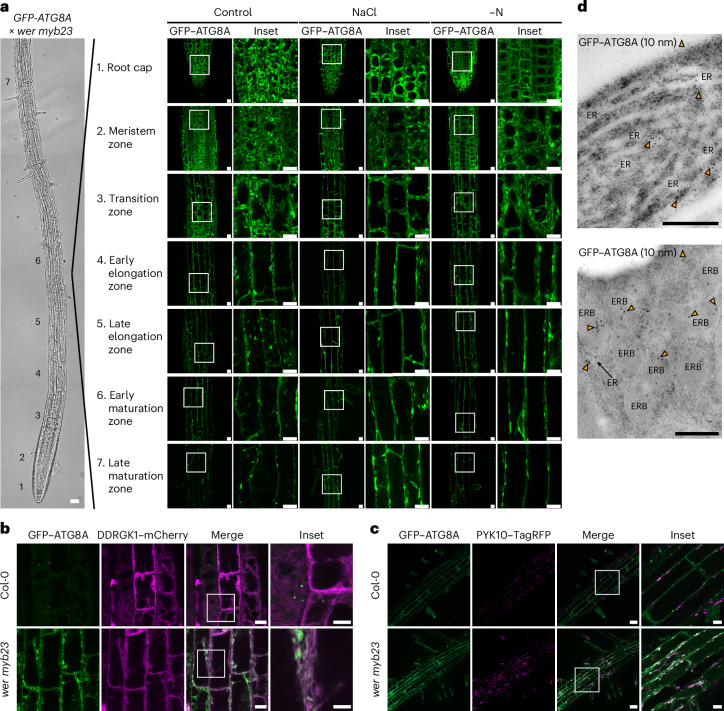
GFP signal derived from GFP–ATG8A localizes predominantly to the ER and ER bodies in *wer* *myb23* mutants. **a**, Confocal microscopy images of cells from different root regions of *wer* *myb23* expressing *ProUBQ10*::*GFP-ATG8A*. Five-day-old *Arabidopsis* seedlings were incubated in control 1/2 MS media (Control) for 3 h, 100 mM NaCl-containing 1/2 MS media (NaCl) for 45 min or nitrogen-deficient 1/2 MS media (−N) for 3 h before imaging. Representative images of three replicates are shown. The areas highlighted by white boxes in the GFP–ATG8A panels are further enlarged and presented in the inset panels. Bright-field scale bar, 50 μm. GFP–ATG8A panel scale bars, 10 μm. Inset scale bars, 10 μm. **b**, Confocal microscopy images of epidermal cells of the root transition zone of Col-0 or *wer* *myb23* co-expressing *ProUBQ10*::*GFP-ATG8A* and *ProUBQ10*::*DDRGK1-mCherry*. Five-day-old *Arabidopsis* seedlings were incubated in 1/2 MS media for 45 min before imaging. Representative images of two replicates are shown. The areas highlighted by white boxes in the merge panels are further enlarged and presented in the inset panels. Scale bars, 10 μm. Inset scale bars, 5 μm. **c**, Confocal microscopy images of epidermal cells of the root elongation zone of Col-0 or *wer* *myb23* co-expressing *ProPYK10*::*PYK10-TagRFP* and *ProUBQ10*::*GFP-ATG8A*. Five-day-old *Arabidopsis* seedlings were incubated in 1/2 MS media for 45 min before imaging. Representative images of three replicates are shown. The areas highlighted by white boxes are further enlarged and presented in the inset panels. Scale bars, 30 μm. Inset scale bars, 10 μm. **d**, Immunogold labelling transmission electron micrographs showing the GFP signal localized to the ER and ER bodies (ERB) in the root epidermal cells of *wer* *myb23*. GFP–ATG8A was labelled with rabbit anti-GFP primary antibody and secondary antibody conjugated to 10-nm gold particles (yellow arrowheads). Scale bars, 500 nm. Representative images from three individual seedlings are shown.

**Extended Data Fig. 3:**
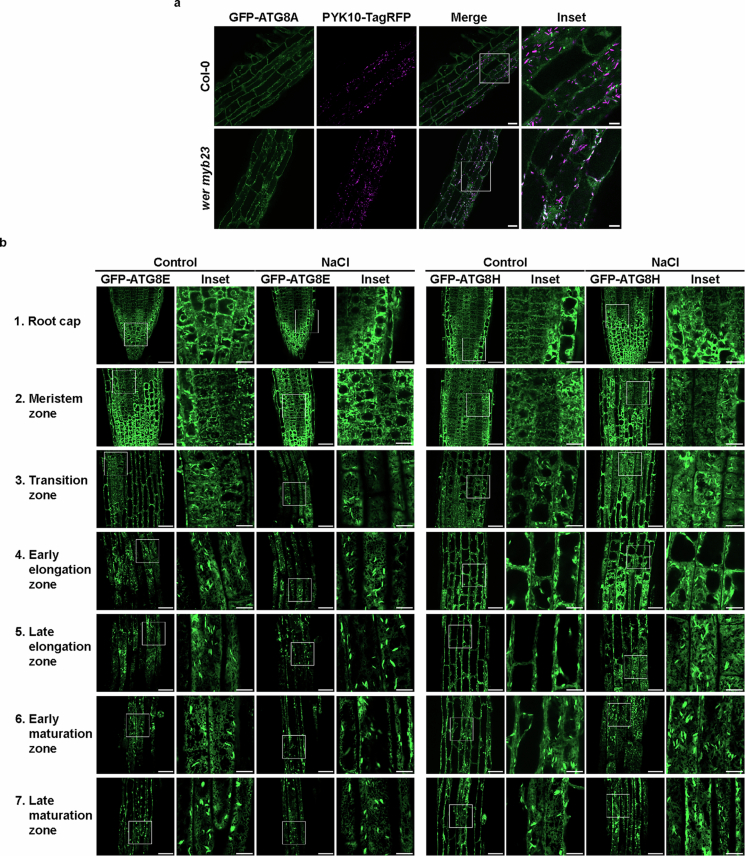
GFP signal derived from GFP-ATG8 localize predominantly to the endoplasmic reticulum and endoplasmic reticulum bodies in *wer myb23* mutants. **a**. Confocal microscopy images of the epidermal cells of root elongation zone of the Arabidopsis wildtype Col-0 or *wer myb23* mutant line co-expressing ProPYK10:PYK10-TagRFP and ProUBQ10:GFP-ATG8A. 5 days old Arabidopsis seedlings were incubated in 100 mM NaCl-containing 1/2 MS media for 45 min before imaging. Representative images of 3 replicates are shown. Area highlighted in the white-boxed region was further enlarged and presented in the inset panel. Scale bars, 30 μm. Inset scale bars, 10 μm. **b**. Confocal microscopy images of cells from different root regions of the Arabidopsis mutant line *wer myb23* expressing ProUBQ10:GFP-ATG8E or ProUBQ10:GFP-ATG8H. 5 days old Arabidopsis seedlings were incubated in either control 1/2 MS media (Control) or 100 mM NaCl-containing 1/2 MS media (NaCl) for 45 min before imaging. Representative images of 2 replicates are shown. Area highlighted in the white-boxed region was further enlarged and presented in the inset panel. Scale bars, 30 μm. Inset scale bars, 10 μm.

**Extended Data Fig. 4:**
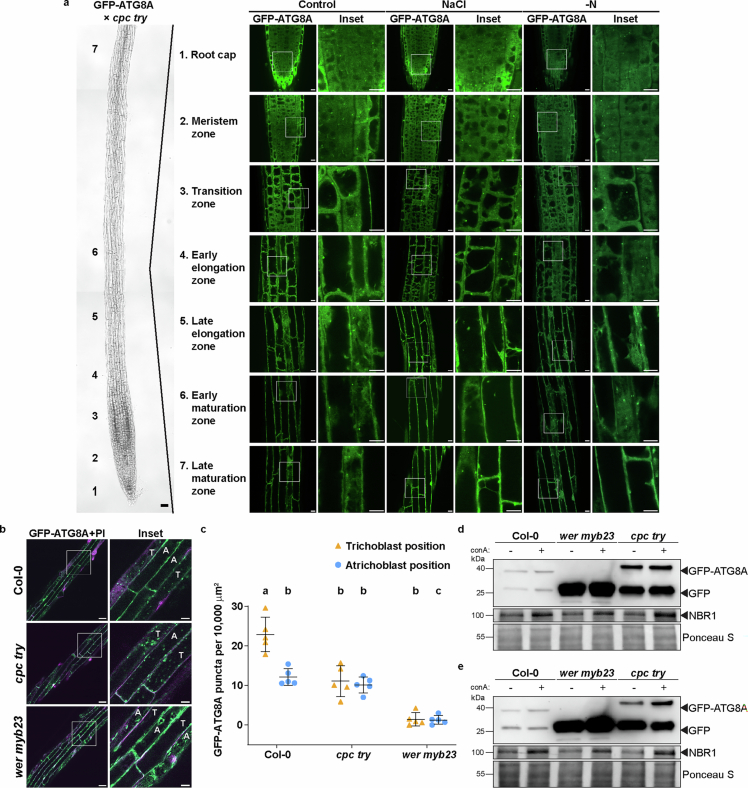
GFP-ATG8A does not localize to the endoplasmic reticulum and endoplasmic reticulum bodies in *cpc* *try* mutants. **a**. Confocal microscopy images of cells from different root regions of the Arabidopsis mutant line *cpc* *try* expressing ProUBQ10:GFP-ATG8A. 5 days old Arabidopsis seedlings were incubated in either control 1/2 MS media (Control) for 3 h, 100 mM NaCl-containing 1/2 MS media (NaCl) for 45 min, or nitrogen-deficient 1/2 MS media ( − N) for 3 h before imaging. Representative images of 3 replicates are shown. Area highlighted in the white-boxed region in the GFP-ATG8A panel was further enlarged and presented in the inset panel. Bright field scale bars, 50 μm. GFP-ATG8A panel scale bars, 10 μm. Inset scale bars, 10 μm. **b**. Confocal microscopy images of trichoblasts and atrichoblasts Col-0, *cpc* *try*, and *wer myb23* expressing ProUBQ10:GFP-ATG8A under NaCl treatment. 5 days old Arabidopsis seedlings were incubated in 1/2 MS media containing 100 mM NaCl for 45 min before imaging. Representative images of 5 replicates are shown. Area highlighted in the white-boxed region in the GFP-ATG8A + PI panel was further enlarged and presented in the inset panel. Scale bars, 30 μm. Inset scale bars, 10 μm. Green color, GFP-ATG8A. Magenta color, propidium iodide dye. T, the trichoblast positions (adjacent to two cortex cells). A, the atrichoblast positions (adjacent to only one cortex cell). **c**. Quantification of the GFP-ATG8A puncta per normalized area (10,000 μm^2^) of the cells at the trichoblast positions and the atrichoblast positions imaged in **b**. Bars indicate the mean ± SD of 5 replicates. Paired repeated measures one-way ANOVA and Fisher’s LSD tests were used to analyze the differences of the number of the GFP-ATG8A puncta between each group. Groups sharing the same letters (a/b/c) are not significantly different, while those with different letters are significantly different. Family-wise significance and confidence level, 0.05 (95% confidence interval). **d-e**. Western blots showing GFP-ATG8A cleavage level and endogenous NBR1 level in wildtype Col-0, *wer myb23* and *cpc try* mutants under control (**d**) or NaCl (**e**) treatment. 7 days old Arabidopsis seedlings were treated in either 1/2 MS ± 1 μM conA (**d**) or 50 mM NaCl-containing 1/2 MS ± 1 μM conA (**e**) liquid media for 12 h. Ten μg of total protein extract of the root sections was loaded and immunoblotted with anti-GFP and anti-NBR1 antibodies. Representative images of 2 replicates are shown. Source data

Notably, *wer* *myb23* exhibited an unexpected autophagic flux pattern, with all root epidermal cells displaying very low flux (Fig. 2b–e and Extended Data Fig. 2e,f). Strikingly, we found that in all root epidermal regions, GFP signal derived from GFP–ATG8A in *wer* *myb23* mutant lines was predominantly localized to the endoplasmic reticulum (ER) and ER bodies under both control and autophagy-inducing conditions (Fig. 3a). To confirm our observations, we performed further confocal colocalization and transmission electron microscopy experiments. Our results showed that GFP signal colocalized with the ER marker DDRGK1–mCherry^27^ and the ER body marker PYK10–TagRFP^28^ (Fig. 3b,c and Extended Data Fig. 3a). In addition, immunogold transmission electron microscopy using anti-GFP antibodies verified that GFP signal was enriched at the ER and ER bodies (Fig. 3d). To test whether other ATG8 isoforms show similar localization patterns, we expressed *GFP-ATG8E* and *GFP-ATG8H* in *wer* *myb23* mutants. GFP signal from both GFP–ATG8E and GFP–ATG8H localized to the ER and ER bodies under both control and autophagy-inducing conditions, similar to GFP–ATG8A (Extended Data Fig. 3b). Since *cpc* *try* also showed lower autophagic flux in trichoblasts, we further compared autophagosome biogenesis in Col-0, *cpc* *try* and *wer* *myb23*. Upon autophagy induction, both *cpc* *try* and *wer* *myb23* mutants accumulated significantly fewer autophagosomes than Col-0. In contrast, GFP signal from GFP–ATG8A did not localize to the ER or ER bodies in *cpc* *try* mutants (Extended Data Fig. 4a–c). Western-blotting-based flux assays further showed that GFP–ATG8A full-length protein was detectable in *cpc* *try* but not in *wer* *myb23*, with only free GFP observed in the latter mutant (Extended Data Fig. 4d,e and Supplementary Fig. 1). These data indicate that autophagosome biogenesis is inhibited in the *wer* *myb23* mutant, probably owing to a failure in producing functional full-length GFP–ATG8 fusion protein, alongside with an aberrant enrichment of free GFP at the ER and ER bodies. Together, these findings suggest that the transcriptional network that underlies root epidermal cell development impinges on autophagosome biogenesis and that cell type identity dictates the higher autophagic flux in trichoblast cells.

### Cell-type-specific modulation of autophagy in trichoblasts

**Fig. 4:**
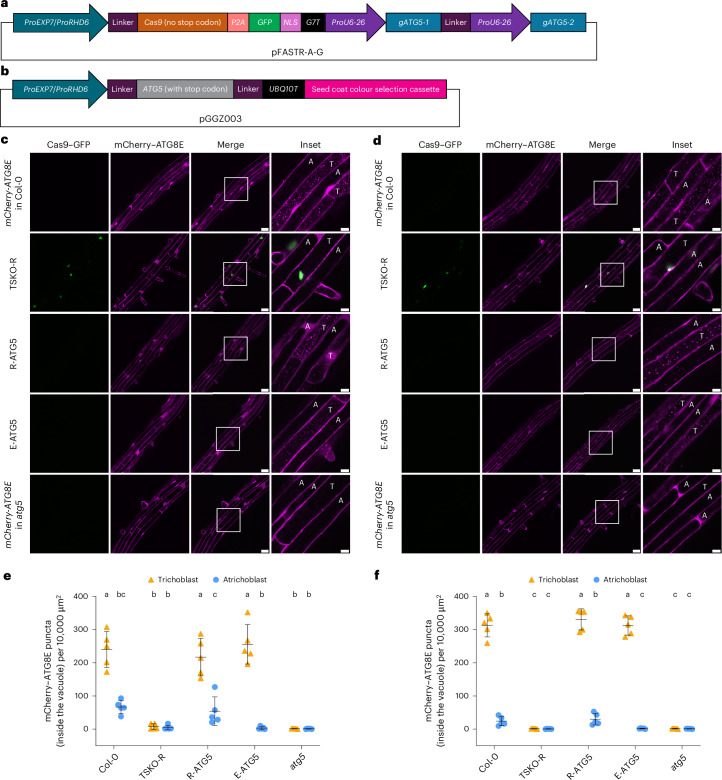
Tissue-specific CRISPR mutagenesis and complementation of *ATG5* confirm higher autophagic flux in trichoblast cells. **a**, Design of the trichoblast-specific *ATG5* CRISPR mutagenesis plasmid expressed in wild-type Col-0 expressing *ProUBQ10*::*mCherry-ATG8E* (*mCherry-ATG8E* in Col-0). **b**, Design of the trichoblast-specific *ATG5* complementation plasmid transformed to complement the autophagy-defective mutant *atg5* expressing *ProUBQ10*::*mCherry-ATG8E* (*mCherry-ATG8E* in *atg5*). **c**,**d**, Confocal microscopy images of trichoblasts and atrichoblasts of *Arabidopsis* lines *mCherry-ATG8E* in Col-0, TSKO-R (*ATG5* mutagenized using *ProRHD6*-driven Cas9), R-ATG5 (the *atg5* mutant complemented with *ProRHD6*-driven *ATG5*), E-ATG5 (the *atg5* mutant complemented with *ProEXP7*-driven *ATG5*) and *mCherry-ATG8E* in *atg5* under control (**c**) or NaCl (**d**) treatment. Five-day-old *Arabidopsis* seedlings were incubated in either control 1/2 MS media containing 2 μM conA for 2 h (**c**) or 1/2 MS media containing 50 mM NaCl + 1 μM conA for 1 h (**d**) before imaging. Representative images of five replicates are shown. The areas highlighted by white boxes in the merge panels are further enlarged and presented in the inset panels. Scale bars, 30 μm. Inset scale bars, 10 μm. T, trichoblast; A, atrichoblast. **e**,**f**, Quantification of mCherry–ATG8E puncta inside the vacuole per normalized area (10,000 μm^2^) of the trichoblasts and atrichoblasts imaged in **c** (**e**) or **d** (**f**). The bars indicate the mean ± s.d. of five replicates. Paired repeated-measures one-way ANOVA and Fisher’s LSD tests were used to analyse the differences in the numbers of mCherry–ATG8E puncta between each group. Groups sharing the same letters (a/b/c) are not significantly different, while those with different letters are significantly different. The family-wise significance and confidence level was 0.05 (95% confidence interval).

**Extended Data Fig. 5:**
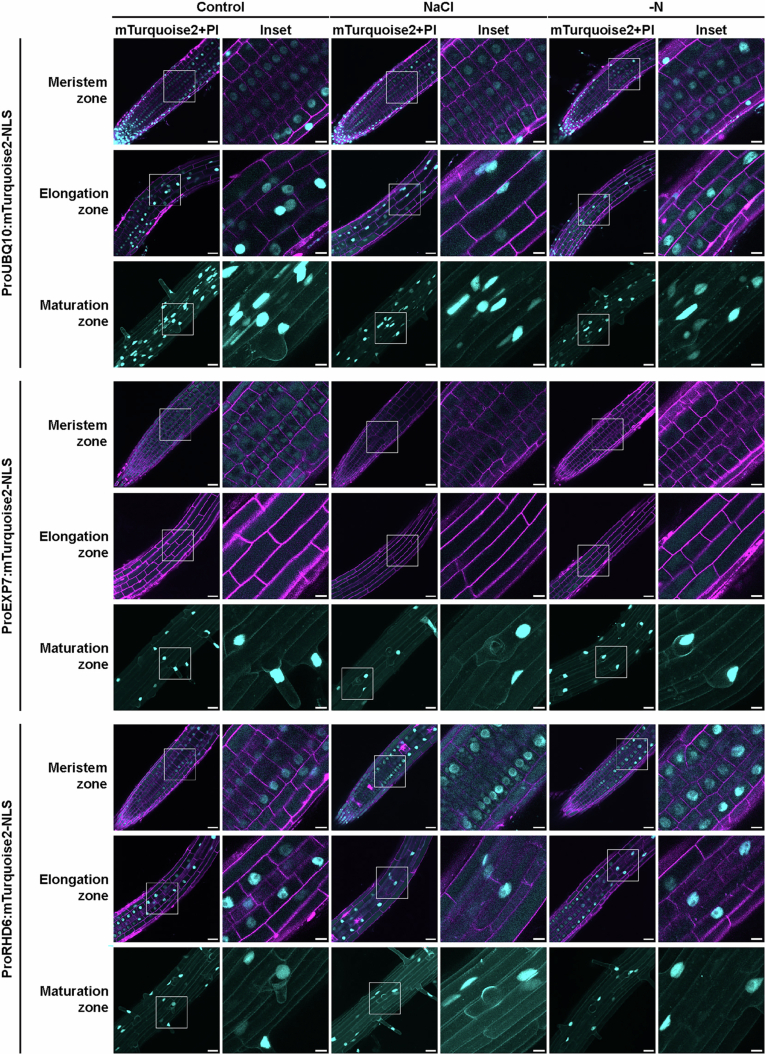
Expression patterns of *UBQ10*, *EXP7* and *RHD6* promoters in *Arabidopsis thaliana* roots. Confocal microscopy images of root epidermal cells of different root regions of Arabidopsis wildtype Col-0 expressing either ProUBQ10:mTurquoise2-NLS, ProEXP7:mTurquoise2-NLS or ProRHD6:mTurquoise2-NLS. 5 days old Arabidopsis seedlings were incubated in either control 1/2 MS media (Control) for 3 h, 100 mM NaCl-containing 1/2 MS media (NaCl) for 45 min, or nitrogen-deficient 1/2 MS media ( − N) for 3 h before imaging. For images showing the maturation zone, an mTurquoise2-single channel Z-stack image was shown. Representative images of 3 replicates are shown. Area highlighted in the white-boxed region in the mTurquoise2+PI panel was further enlarged and presented in the inset panel. Scale bars, 30 μm. Inset scale bars, 10 μm. Cyan color, mTurquoise2-NLS. Magenta color, propidium iodide dye. NLS, nuclear location signal peptide.

**Extended Data Fig. 6:**
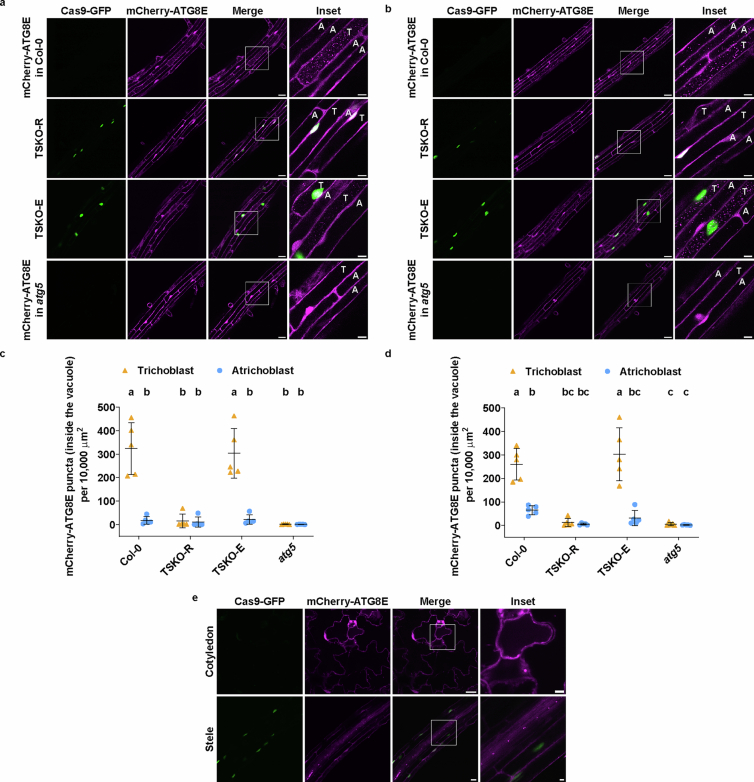
Examination of autophagy dynamics in tissue specific CRISPR mutagenesis lines. **a-d**. ***RHD6*** **promoter is more efficient in disrupting autophagic flux in trichoblast cells compared to** ***EXP7*** **promoter. a-b**. Confocal microscopy images of trichoblasts and atrichoblasts in the root maturation zone of Arabidopsis lines mCherry-ATG8E in Col-0, TSKO-R (*ATG5* mutagenized using *ProRHD6*-driven Cas9), TSKO-E (*ATG5* mutagenized using *ProEXP7*-driven Cas9) and mCherry-ATG8E in atg5 under control (**a**) or (**b**) treatment. 5 days old Arabidopsis seedlings were incubated in either 1/2 MS media containing 2 μM concanamycin A for 2 h (**a**) or 1/2 MS media containing 50 mM NaCl + 1 μM concanamycin A for 1 h (**b**) before imaging. Representative images of 5 replicates are shown. Area highlighted in the white-boxed region in the merge panel was further enlarged and presented in the inset panel. Scale bars, 30 μm. Inset scale bars, 10 μm. T, trichoblasts. A, atrichoblasts. **c-d**. Quantification of the mCherry-ATG8E puncta inside the vacuole per normalized area (10,000 μm^2^) of the cells of the trichoblasts and atrichoblasts imaged in **a** (**c**) or **b** (**d**). Bars indicate the mean ± SD of 5 replicates. Paired repeated measures one-way ANOVA and Fisher’s LSD tests were used to analyze the differences of the number of the mCherry-ATG8E puncta between each group. Groups sharing the same letters (a/b/c) are not significantly different, while those with different letters are significantly different. Family-wise significance and confidence level, 0.05 (95% confidence interval). **e. Normal autophagosome structures can be observed in cells that do not express Cas9 in TSKO-R lines**. Confocal microscopy images of cells of cotyledon epidermis and stele regions of TSKO-R. 5 days old Arabidopsis seedlings were incubated in control 1/2 MS media for 45 min before imaging. Representative images of 3 replicates are shown. Area highlighted in the white-boxed region in the merge panel was further enlarged and presented in the inset panel. Scale bars, 20 μm. Inset scale bars, 5 μm.

To further investigate the physiological significance of the differential autophagic activity between trichoblasts and atrichoblasts, we established two genetic approaches to modulate autophagic flux in trichoblasts. First, using the trichoblast-specific promoters *ProEXP7* and *ProRHD6* (Extended Data Fig. 5) and tissue-specific CRISPR knockout (CRISPR-TSKO) technology^29^, we aimed at blocking autophagy specifically in trichoblasts. We expressed *ProEXP7*-driven and *ProRHD6*-driven Cas9 to knock out *ATG5* in Col-0 expressing *ProUBQ10*::*mCherry-ATG8E* (*mCherry-ATG8E* in Col-0), generating TSKO-E (*ProEXP7*-driven) and TSKO-R (*ProRHD6-*driven) lines (Fig. 4a). Interestingly, under both control and NaCl stress conditions, only TSKO-R exhibited a significant reduction in mCherry–ATG8E-marked autophagic flux in trichoblasts in the maturation zone, while TSKO-E maintained autophagic flux levels similar to Col-0 (Extended Data Fig. 6a–d). Given that *ProEXP7* is expressed exclusively in the trichoblasts of root maturation zone, whereas *ProRHD6* is also expressed in the trichoblasts of root meristematic and elongation zones (Extended Data Fig. 5), we hypothesized that the pre-existing ATG5 is stable enough to reach maturation-zone cells. We also confirmed that TSKO-R did not exhibit reduced autophagic flux in cells that do not express *RHD6* such as the stele and cotyledon epidermis (Extended Data Fig. 6e), indicating that autophagy remained intact in non-*ProRHD6*-expressing regions. On the basis of these results, we selected TSKO-R for further studies.

In parallel, we generated *Arabidopsis* lines in which autophagy was specifically rescued in trichoblasts. We expressed *ProEXP7*-driven and *ProRHD6*-driven *ATG5* in *Arabidopsis* *atg5* mutants expressing *ProUBQ10*::*mCherry-ATG8E* (*mCherry-ATG8E* in *atg5*), creating the complementation lines R-ATG5 (*ProRHD6*-driven) and E-ATG5 (*ProEXP7*-driven) (Fig. 4b). Subsequently, we compared the mCherry–ATG8E-marked autophagic flux in Col-0, TSKO-R, R-ATG5, E-ATG5 and *atg5* under control and NaCl stress conditions. TSKO-R lost the autophagic flux difference between trichoblasts and atrichoblasts, resembling the *atg5* mutant. In contrast, both R-ATG5 and E-ATG5 restored the autophagic flux difference to levels comparable to *mCherry-ATG8E* in Col-0 (Fig. 4c–f). These results demonstrate that TSKO-R, R-ATG5 and E-ATG5 allow us to study the physiological relevance of higher autophagic flux in trichoblast cells.

### Elevated trichoblast autophagy contributes to salt stress tolerance

**Fig. 5:**
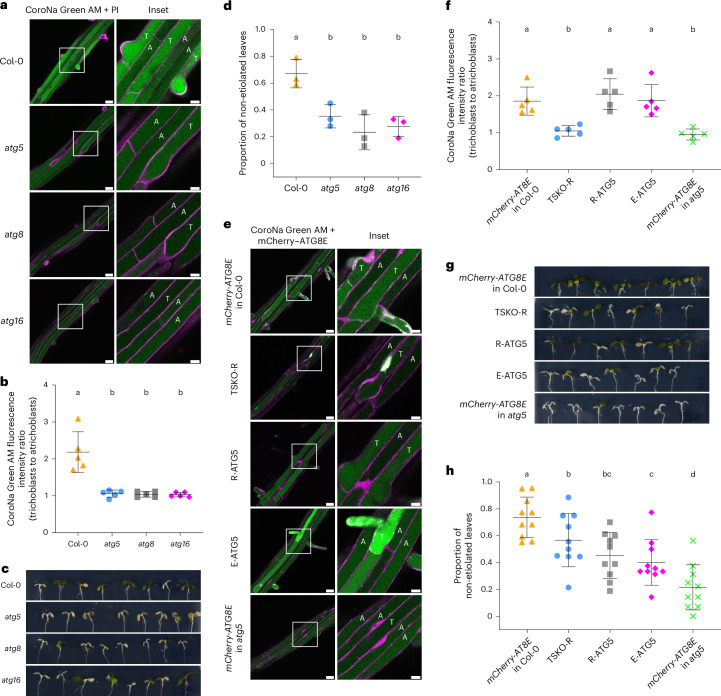
Autophagy regulates sodium accumulation in the vacuoles of trichoblast cells and is essential for NaCl stress tolerance. **a**, Confocal microscopy images showing sodium ion accumulation in the vacuoles of trichoblasts and atrichoblasts in the root maturation zone of *Arabidopsis* wild-type Col-0 and the autophagy-defective mutants *atg5*, *atg8* and *atg16*. Five-day-old *Arabidopsis* seedlings were incubated in control 1/2 MS media for 30 min and were subsequently incubated in control 1/2 MS media containing 2 μM CoroNa Green AM for 30 min before imaging. Representative images of five replicates are shown. The areas highlighted by white boxes in the CoroNa Green AM + PI panels are further enlarged and presented in the inset panels. Scale bars, 30 μm. Inset scale bars, 10 μm. Green indicates CoroNa Green AM sodium indicator; magenta indicates PI dye. T, trichoblast; A, atrichoblast. **b**, Quantitative analysis of the vacuolar CoroNa Green AM fluorescence intensity ratio between trichoblasts and atrichoblasts of the *Arabidopsis* seedlings imaged in **a**. The bars indicate the mean ± s.d. of five replicates. Paired repeated-measures one-way ANOVA and Fisher’s LSD tests were performed to analyse the significance of the CoroNa Green AM fluorescence intensity ratio differences between each group. Groups sharing the same letters (a/b) are not significantly different, while those with different letters are significantly different. The family-wise significance and confidence level was 0.05 (95% confidence interval). **c**, Phenotypic characterization of the seedlings of *Arabidopsis* wild-type Col-0 and the autophagy-defective mutants *atg5*, *atg8* and *atg16* under NaCl stress treatment. Representative images of three replicates are shown. **d**, Quantitative analysis of the proportion of non-etiolated leaves of the *Arabidopsis* seedlings imaged in **c**. The bars indicate the mean ± s.d. of three replicates. Paired repeated-measures one-way ANOVA and Fisher’s LSD tests were performed to analyse the significance of the proportion differences between each group. Groups sharing the same letters (a/b) are not significantly different, while those with different letters are significantly different. The family-wise significance and confidence level was 0.05 (95% confidence interval). **e**, Confocal microscopy images showing the sodium ion concentrations in the vacuoles of trichoblasts and atrichoblasts in the root maturation zone of *Arabidopsis* lines *mCherry-ATG8E* in Col-0, TSKO-R, R-ATG5, E-ATG5 and *mCherry-ATG8E* in *atg5*. Five-day-old *Arabidopsis* seedlings were incubated in control 1/2 MS media for 30 min and were subsequently incubated in control 1/2 MS media containing 2 μM CoroNa Green AM for 30 min before imaging. Representative images of five replicates are shown. The areas highlighted by white boxes in the CoroNa Green AM + mCherry–ATG8E panels are further enlarged and presented in the inset panels. Scale bars, 30 μm. Inset scale bars, 10 μm. Green indicates CoroNa Green AM sodium indicator (and the nuclear signals of Cas9–GFP in TSKO-R); magenta indicates mCherry–ATG8E. T, trichoblast; A, atrichoblast. **f**, Quantitative analysis of the vacuolar CoroNa Green AM fluorescence intensity ratio between trichoblasts and atrichoblasts of the *Arabidopsis* seedlings imaged in **e**. The bars indicate the mean ± s.d. of five replicates. Paired repeated-measures one-way ANOVA and Fisher’s LSD tests were performed to analyse the significance of the CoroNa Green AM fluorescence intensity ratio differences between each group. Groups sharing the same letters (a/b) are not significantly different, while those with different letters are significantly different. The family-wise significance and confidence level was 0.05 (95% confidence interval). **g**, Phenotypic characterization of the seedlings of *Arabidopsis* lines *mCherry-ATG8E* in Col-0, TSKO-R, R-ATG5, E-ATG5 and *mCherry-ATG8E* in *atg5* under NaCl stress treatment. Representative images of ten replicates are shown. **h**, Quantitative analysis of the proportion of non-etiolated leaves of the *Arabidopsis* seedlings imaged in **g**. The bars indicate the mean ± s.d. of ten replicates. Paired repeated-measures one-way ANOVA and Fisher’s LSD tests were performed to analyse the significance of the proportion differences between each group. Groups sharing the same letters (a/b/c/d) are not significantly different, while those with different letters are significantly different. The family-wise significance and confidence level was 0.05 (95% confidence interval).

**Extended Data Fig. 7:**
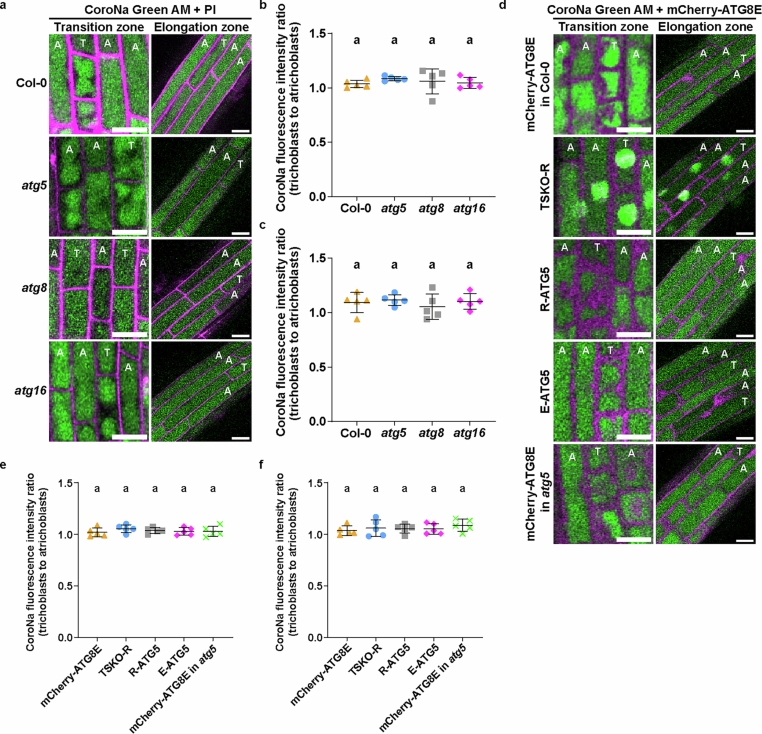
Sodium ion levels in the vacuoles of trichoblasts and atrichoblasts in the transition zone and the elongation zone are not significantly different. **a**. Confocal microscopy images showing sodium ion concentrations in the vacuoles of trichoblasts and atrichoblasts in the transition zone and the elongation zone of Arabidopsis wildtype Col-0 and the autophagy-defective mutants *atg5*, *atg8* and *atg16* indicated by CoroNa Green AM staining. 5 days old Arabidopsis seedlings were incubated in control 1/2 MS media for 30 min and were subsequently incubated in control 1/2 MS media containing 2 μM CoroNa Green AM for 30 min before imaging. Representative images of 5 replicates are shown. Scale bars, 20 μm. Green color, CoroNa Green AM sodium indicator. Magenta color, propidium iodide dye. T, trichoblasts. A, atrichoblasts. **b-c**. Quantitative analysis of the vacuolar CoroNa Green AM fluorescence intensity ratio between trichoblasts and atrichoblasts at the transition zone (**b**) or elongation zone (**c**) of the Arabidopsis seedlings imaged in **a**. Bars indicate the mean ± SD of 5 replicates. Paired repeated measures one-way ANOVA and Fisher’s LSD tests were performed to analyze the significance of the CoroNa Green AM fluorescence intensity ratio differences between each group. Groups sharing the same letter ‘a’ are not significantly different. Family-wise significance and confidence level, 0.05 (95% confidence interval). **d**. Confocal microscopy images showing sodium ion concentrations in the vacuoles of trichoblasts and atrichoblasts in the transition zone and the elongation zone of Arabidopsis lines mCherry-ATG8E in Col-0, TSKO-R, R-ATG5, E-ATG5 and mCherry-ATG8E in *atg5* indicated by CoroNa Green AM staining. 5 days old Arabidopsis seedlings were incubated in control 1/2 MS media for 30 min and were subsequently incubated in control 1/2 MS media containing 2 μM CoroNa Green AM for 30 min before imaging. Representative images of 5 replicates are shown. Scale bars, 20 μm. Green color, CoroNa Green AM sodium indicator (and the nuclear signals of Cas9-GFP in TSKO-R). Magenta color, mCherry-ATG8E. T, trichoblasts. A, atrichoblasts. **e-f**. Quantitative analysis of the vacuolar CoroNa Green AM fluorescence intensity ratio between trichoblasts and atrichoblasts in the transition zone (**e**) or elongation zone (**f**) of the Arabidopsis seedlings imaged in **d**. Bars indicate the mean ± SD of 5 replicates. Paired repeated measures one-way ANOVA and Fisher’s LSD tests were performed to analyze the significance of the CoroNa Green AM fluorescence intensity ratio differences between each group. Groups sharing the same letter ‘a’ are not significantly different. Family-wise significance and confidence level, 0.05 (95% confidence interval).

**Extended Data Fig. 8:**
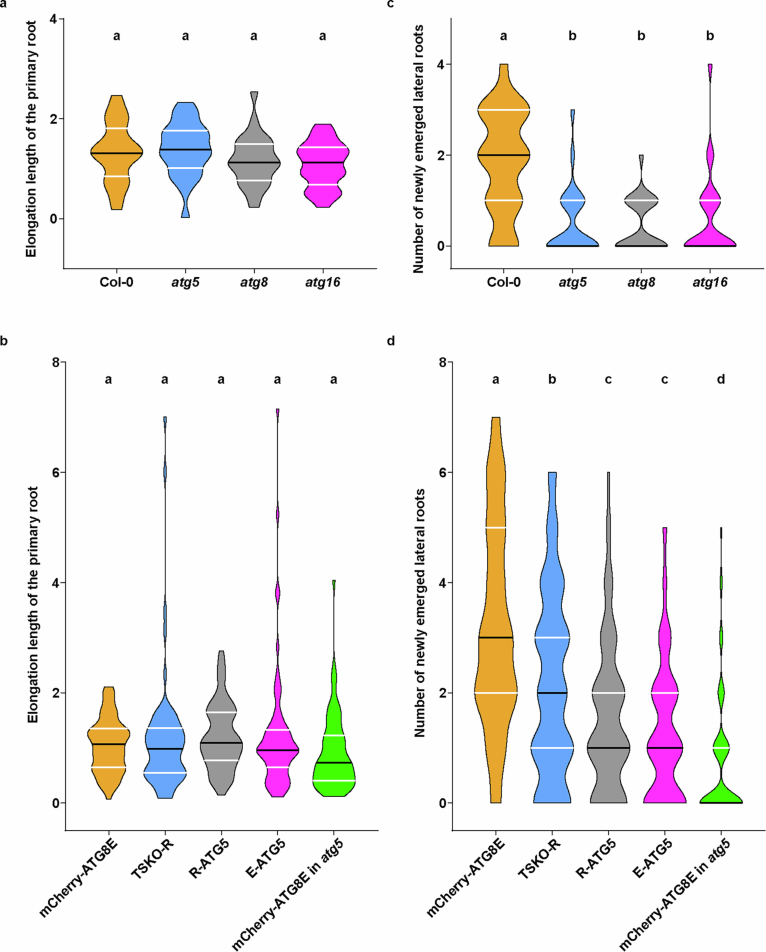
Higher autophagic flux in trichoblasts affects the emergence of lateral roots rather than primary root growth. **a-b**. Quantitative analysis of primary root length of Arabidopsis seedlings imaged in Fig. 5c (**a**, n = 24) or Fig. 5g (**b**, n = 70). Repeated measures one-way ANOVA and Tukey tests were performed to analyze the significance of the primary root elongation difference between each group. Groups sharing the same letter ‘a’ are not significantly different. Family-wise significance and confidence level, 0.05 (95% confidence interval). Black lines indicate the median. White lines, 25% and 75% quartiles. **c-d**. Quantitative analysis of the number of newly emerged lateral roots of Arabidopsis seedlings imaged in Fig. 5c (**a**, n = 24) or Fig. 5g (**b**, n = 70). Unpaired Brown-Forsythe and Welch ANOVA tests and two-sided unpaired t tests with Welch’s correction (for multiple comparison) were performed to analyze the significance of the number of newly emerged lateral roots between each group, n = 24. Groups sharing the same letters (a/b/c/d) are not significantly different, while those with different letters are significantly different. Family-wise significance and confidence level, 0.05 (95% confidence interval). Black lines indicate the median. White lines, 25% and 75% quartiles. Note that the median and 25% quartile lines of *atg5*, *atg8*, and *atg16* were all at the base line (y = 0), and the median line of mCherry-ATG8E in *atg5*, the 25% quartile lines of R-ATG5, E-ATG5 and mCherry-ATG8E in *atg5* were all at the base line (y = 0).

Next, we set out to explore the physiological relevance of higher autophagic flux in trichoblast cells. A previous study showed that autophagy is required for sodium ion accumulation in the vacuoles of root meristem cells^30^. This finding inspired us to check whether higher flux in trichoblast cells is correlated with the accumulation of sodium in trichoblast vacuoles. First, we measured sodium ion concentrations in the vacuoles of epidermal cells of Col-0 and three autophagy-deficient mutants (*atg5*, *atg8* and *atg16*), using the CoroNa Green AM staining method^30,31^. At the transition zone and elongation zone, trichoblasts and adjacent atrichoblasts had similar vacuolar sodium levels (Extended Data Fig. 7a–c). In contrast, in wild-type plants, trichoblasts in the root maturation zone exhibited significantly higher vacuolar sodium ion concentrations than atrichoblasts (CoroNa Green AM fluorescence ratios >1.5), whereas all autophagy-defective mutants lost this difference (CoroNa Green AM fluorescence ratios around 1) (Fig. 5a,b). This prompted us to hypothesize that sodium accumulation in the vacuoles of trichoblasts in the root maturation zone could help plants tolerate NaCl stress. To test our hypothesis, we grew seeds of Col-0 and autophagy-deficient mutant lines on normal half-strength Murashige and Skoog (1/2 MS) media for six days and then transferred them to 1/2 MS media supplemented with 150 mM NaCl for four days. After four days of salt treatment, Col-0 seedlings displayed significantly higher survival rates (measured as the proportion of non-etiolated leaves) than the autophagy-deficient lines (Fig. 5c,d). In addition, although the primary root growth showed no difference between Col-0 and the autophagy-defective mutants, the wild-type plants had more newly emerged lateral roots (Extended Data Fig. 8a,c).

We then tested whether higher flux in trichoblast cells contributes to NaCl stress tolerance, using the tools we established. The CoroNa Green AM staining results revealed that TSKO-R, which lacks autophagic flux differences between trichoblasts and atrichoblasts, also specifically lost the sodium ion concentration difference in the root maturation zone (Fig. 5e,f and Extended Data Fig. 7d,f). Conversely, the complementation lines R-ATG5 and E-ATG5, which restored autophagic flux differences, also re-established the sodium ion concentration difference between trichoblasts and atrichoblasts (Fig. 5e,f). Given that only a subset of cells in TSKO-R lost autophagy and only a subset in R-ATG5 and E-ATG5 regained autophagy, we predicted that these lines would exhibit intermediate survival rates and intermediate numbers of newly emerged lateral roots. Indeed, the survival rates and the numbers of newly emerged lateral roots of the TSKO-R, R-ATG5 and E-ATG5 lines were significantly lower than those of wild-type Col-0 but significantly higher than those of *atg5* (Fig. 5g,h and Extended Data Fig. 8d). Collectively, these results demonstrate that autophagy mediates sodium ion sequestration in trichoblast vacuoles, thereby contributing to NaCl stress tolerance in *Arabidopsis* seedlings.

### Autophagy is essential for reactive oxygen species scavenging in trichoblasts

**Extended Data Fig. 9:**
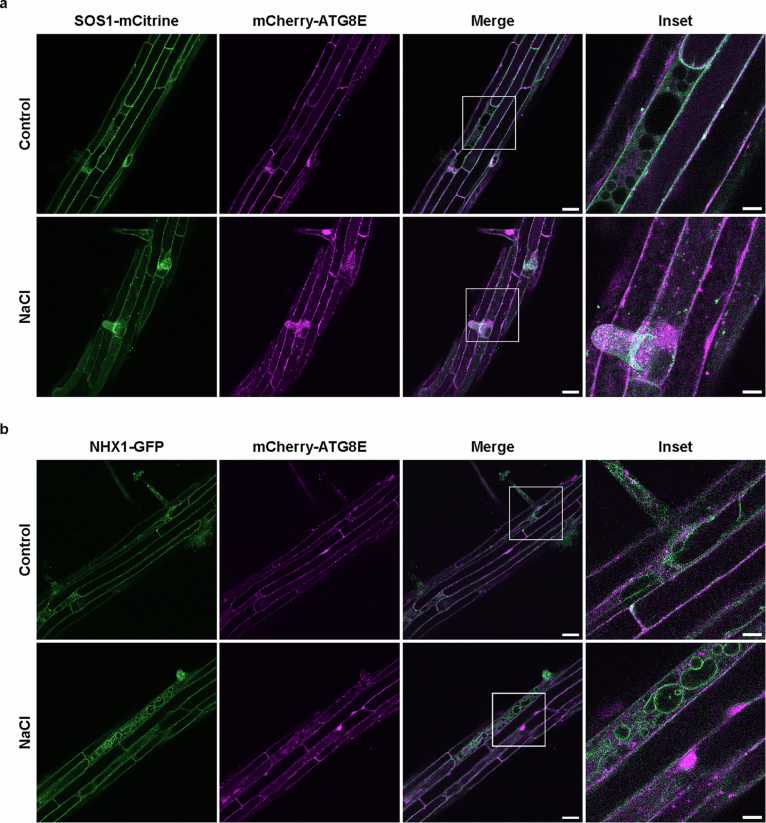
The Na^+^/H^+^ antiporter SOS1 and NHX1 do not colocalize with the ATG8E-labeled autophagosomes. **a**. Confocal microscopy images of the epidermal cells in the root maturation zone of Arabidopsis expressing ProSOS1:SOS1-mCitrine and ProUBQ10:mCherry-ATG8E. 5 days old Arabidopsis seedlings were incubated in either control 1/2 MS media (Control) or 50 mM NaCl-containing 1/2 MS media (NaCl) for 45 min before imaging. Representative images of 2 replicates are shown. Area highlighted in the white-boxed region was further enlarged and presented in the inset panel. Scale bars, 30 μm. Inset scale bars, 10 μm. **b**. Confocal microscopy images of the epidermal cells in the root maturation zone of Arabidopsis expressing ProUBQ10:NHX1-GFP and ProUBQ10:mCherry-ATG8E. 5 days old Arabidopsis seedlings were incubated in either control 1/2 MS media (Control) or 50 mM NaCl-containing 1/2 MS media (NaCl) for 45 min before imaging. Representative images of 2 replicates are shown. Area highlighted in the white-boxed region was further enlarged and presented in the inset panel. Scale bars, 30 μm. Inset scale bars, 10 μm.

We next sought to dissect the molecular link between salt stress tolerance and autophagy. Although the precise regulatory mechanism remains to be fully elucidated, membrane transporters such as Salt Overly Sensitive 1 (SOS1) and Na^+^/H^+^ Exchanger 1 (NHX1) maintain ion homoeostasis and play pivotal roles in salt stress tolerance^32–36^. We thus hypothesized that autophagy may modulate the levels of these transporters in trichoblasts by selectively degrading SOS1 and NHX1 proteins. To test this hypothesis, we generated *Arabidopsis* lines co-expressing *SOS1-mCitrine* or *NHX1-GFP* with *mCherry-ATG8E* and examined their subcellular localization. Confocal analyses revealed that neither SOS1 nor NHX1 colocalized with mCherry–ATG8E under either control conditions or NaCl treatment (Extended Data Fig. 9).

**Fig. 6:**
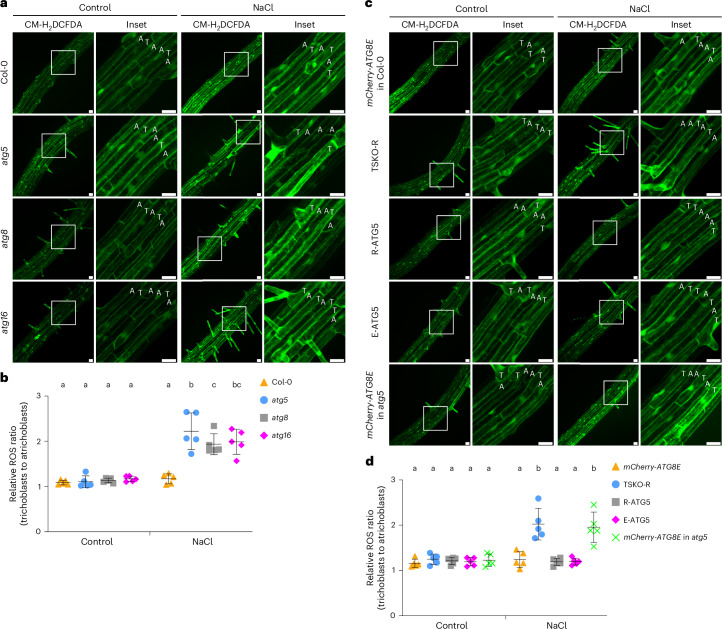
Relative ROS levels in trichoblasts and atrichoblasts are correlated with the levels of autophagic flux. **a**, Confocal microscopy images showing the relative ROS levels in the trichoblasts and atrichoblasts of *Arabidopsis* wild-type Col-0 and the autophagy-defective mutants *atg5*, *atg8* and *atg16*. Five-day-old *Arabidopsis* seedlings were incubated in either 1/2 MS (control) or 1/2 MS + 50 mM NaCl (NaCl) liquid media for 45 min and were subsequently stained with 1 μM CM-H_2_DCFDA for 1 min before imaging. Representative images of five replicates are shown. The areas highlighted by white boxes in the CM-H_2_DCFDA panels are further enlarged and presented in the inset panels. Scale bars, 30 μm. Inset scale bars, 30 μm. T, trichoblast; A, atrichoblast. **c**, Confocal microscopy images showing the relative ROS levels in the trichoblasts and atrichoblasts of *Arabidopsis* lines *mCherry-ATG8E* in Col-0, TSKO-R, R-ATG5, E-ATG5 and *mCherry-ATG8E* in *atg5*. Five-day-old *Arabidopsis* seedlings were incubated in either 1/2 MS (control) or 1/2 MS + 50 mM NaCl (NaCl) liquid media for 45 min and were subsequently stained with 1 μM CM-H_2_DCFDA for 1 min before imaging. Representative images of five replicates are shown. The areas highlighted by white boxes in the CM-H_2_DCFDA panels are further enlarged and presented in the inset panels. Scale bars, 30 μm. Inset scale bars, 30 μm. **b**,**d**, Quantitative analysis of the relative ROS ratio between trichoblasts and atrichoblasts of the *Arabidopsis* seedlings imaged in **a** (**b**) or **c** (**d**). The bars indicate the mean ± s.d. of five replicates. Paired repeated-measures two-way ANOVA and Fisher’s LSD tests were performed to analyse the significance of the CM-H_2_DCFDA fluorescence intensity ratio differences between each group. Groups sharing the same letter (a/b/c) are not significantly different, while those with different letters are significantly different. The family-wise significance and confidence level was 0.05 (95% confidence interval).

Another strategy employed by plants to tolerate salt stress is reactive oxygen species (ROS) scavenging^37^. Prior work has demonstrated that autophagy-defective mutants have elevated ROS levels in *Arabidopsis*^30^, suggesting that autophagy contributes to ROS homoeostasis. This prompted us to hypothesize that higher flux in trichoblasts could be due to higher ROS levels in these cells. To test this hypothesis, we quantified ROS levels in trichoblasts and atrichoblasts in the root maturation zone of wild-type Col-0 and the autophagy-defective mutants *atg5*, *atg8* and *atg16* using CM-H_2_DCFDA staining^38,39^. Under control conditions, the ratio of ROS levels in trichoblasts versus atrichoblasts was ~1.1 across all genotypes (Fig. 6a,b). In contrast, following NaCl stress, autophagy-defective mutants accumulated significantly higher ROS levels in trichoblasts than in atrichoblasts, a phenotype that was not observed in wild-type Col-0 (Fig. 6a,b). These observations indicate that the impairment of autophagy leads to ROS accumulation in trichoblasts. To further corroborate this link, we measured ROS levels in the tissue-specific autophagy mutant lines. TSKO-R lines, where autophagy is specifically blocked in trichoblasts, mimicked the ROS accumulation phenotype of *atg5*, *atg8* and *atg16* mutants (Fig. 6c,d). Conversely, cell-type-specific complementation of the autophagy pathway in trichoblasts established in R-ATG5 and E-ATG5 lines prevented ROS accumulation in trichoblasts (Fig. 6c,d). Collectively, these findings demonstrate that enhanced autophagic activity in trichoblasts mediates ROS scavenging, thereby contributing to salt stress tolerance.

**Extended Data Fig. 10:**
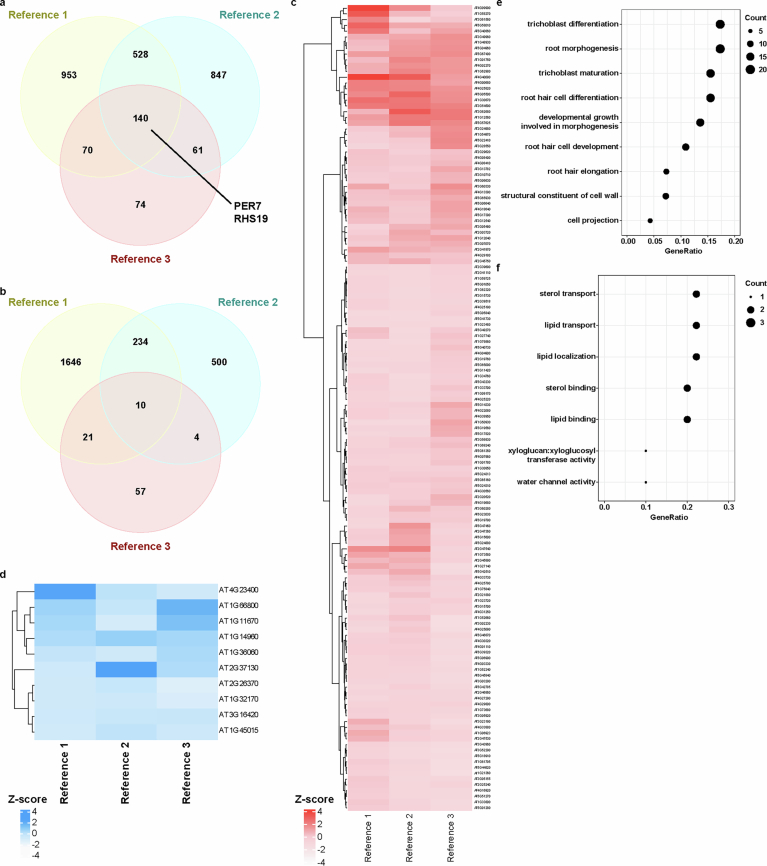
Comparative analysis of trichoblast and atrichoblast gene sets across three single-cell RNA-seq datasets. **a-b**. Venn diagram showing the number of genes that are highly expressed in either trichoblasts (**a**) or atrichoblasts (**b**) of Arabidopsis wildtype Col-0 from three published single-cell RNA-seq datasets^40–42^. **c-d**. Heatmap showing the genes that are highly expressed in trichoblasts (**c**) or atrichoblasts (**d**) in all three reference datasets. **e-f**. Bubble plots showing the gene ontology enrichment analysis of the highly expressed genes identified in **c** (**e**) and **d** (**f**). Gene ratio was calculated as the count number divided by the size of the datasets.

To investigate the molecular mechanism leading to elevated autophagic flux in trichoblasts, we hypothesized that *ATG* genes or other genes that are involved in autophagy regulation could have higher expression levels in trichoblasts than in atrichoblasts. Analysis of three published single-cell RNA sequencing (RNA-seq) datasets revealed 140 genes that are expressed at higher levels in trichoblasts and 10 genes that are more induced in atrichoblasts^40–42^ (Extended Data Fig. 10 and Supplementary Tables 1 and 2). The majority of these 140 trichoblast-enriched genes were associated with trichoblast differentiation and root hair morphogenesis (Extended Data Fig. 10e and Supplementary Tables 1 and 2). *ATG* genes were not differentially expressed (Supplementary Tables 1 and 2). Intriguingly, two peroxidases, PEROXIDASE 7 and Root Hair Specific 19, ranked among the top 20 trichoblast-induced genes across all datasets, implying that the maintenance of ROS homoeostasis is pivotal for trichoblast development and root hair formation (Extended Data Fig. 10e and Supplementary Tables 1 and 2).

## Discussion

Our study uncovers a critical role for cell-type-specific autophagy in trichoblasts, linking higher autophagic flux to sodium ion sequestration and salt stress tolerance in *A.* *thaliana* (Figs. 1 and 5). Under both control and stress conditions, trichoblasts exhibit significantly higher autophagic activity than atrichoblasts, and this difference is essential for vacuolar sodium accumulation and plant survival under salinity.

We envision three scenarios that may explain autophagy-mediated sodium sequestration in the vacuole. First, autophagy could support sodium compartmentalization via modulating the turnover or activity of sodium transporters. Previous studies in mammalian cells have shown that ion channels could modulate autophagy by ion fluxes in and out of lysosomes and that they themselves could be modulated by autophagic recycling^43^. Although SOS1 and NHX1 do not colocalize with autophagosome markers (Extended Data Fig. 9), other transporters could be regulated by autophagy. Alternatively, metalloproteins that can carry sodium ions in bulk could be delivered to the vacuole via autophagy. This is akin to ferritinophagy that is mediated by the NCOA4 selective autophagy receptor^44^. NCOA4 selectively binds ferritin and delivers ferritin molecules to the lysosomes to release iron^45^. Similar to ferritinophagy, sodium-binding metalloproteins could be targeted by autophagy to rapidly deliver sodium ions to the vacuole and prevent cytotoxicity. These two scenarios focus on the degradative function of autophagy. The third possibility is that higher autophagic flux could improve cellular homoeostasis, enabling trichoblasts to maintain ROS homoeostasis more effectively under stress. This is consistent with elevated ROS levels in TSKO-R and autophagy-deficient mutants under NaCl stress (Fig. 6). Further studies that aim to uncover autophagic cargo under salt stress or mechanistically link ROS accumulation to autophagy will reveal how autophagy contributes to salt stress tolerance in plants.

The differential autophagic flux between trichoblasts and atrichoblasts originates during cell fate specification at the meristematic zone, as evidenced by epidermal mutants (Fig. 2 and Extended Data Fig. 2e,f). Mutants disrupting early trichoblast/atrichoblast identity (*cpc* *try* and *wer* *myb23*) abolished autophagic differences, while those retaining cell fate (*rhd6* *rsl1* and *gl2*) preserved them. This suggests that developmental programs dictating trichoblast/atrichoblast identity directly regulate autophagy. Transcription factors or signalling molecules common to both pathways may mediate this crosstalk. Mislocalization of the GFP signal to the ER and ER bodies in *wer* *myb23* mutants further highlights this interplay, implying that cell fate regulators impinge on autophagosome biogenesis machinery. An intriguing observation is that concanamycin A (conA) treatment increases NBR1 protein levels in *wer* *myb23* mutants, despite no detectable flux detected with GFP–ATG8 fusions (Extended Data Fig. 4d,e and Supplementary Fig. 1). This probably suggests that conA treatment could induce the expression of NBR1 and serves as a cautionary note to assess autophagic flux using orthogonal assays. Nevertheless, the entrapment of GFP–ATG8 fusions in *wer* *myb23* mutants opens new avenues to explore how developmental signalling could spatially regulate autophagy.

Overall, our findings underscore the importance of studying autophagy at cellular resolution. Whole-organism approaches may overlook critical tissue- or cell-type-specific dynamics, as demonstrated by the stark contrast between trichoblasts and atrichoblasts. Readily available high-resolution tools such as CRISPR-TSKO, single-cell omics and live-cell imaging allow us to move from whole-organism to cell-type-specific autophagy studies and open the door to address a fundamental yet unanswered question in cell biology: how autophagy responses in different cell types are coordinated to establish an organismal homeostatic response.

By manipulating autophagy specifically in trichoblasts using CRISPR-TSKO and ATG5 complementation lines, we demonstrated its necessity and sufficiency for sodium accumulation and salt tolerance (Fig. 5). Disrupting autophagy in trichoblasts (TSKO-R) abolished sodium differences and reduced survival under salt stress, while restoring it (R-ATG5 and E-ATG5) reinstated both. These results suggest that enhancing autophagy in stress-responsive cell types, such as trichoblasts, could improve crop resilience without compromising growth. For agriculture, this strategy could mitigate yield losses in saline soils, particularly in staple crops where root hair function is pivotal for nutrient uptake.

## Methods

### Plant material and cloning procedure

All *A.* *thaliana* lines used in this study are listed in Supplementary Table 3. All the plasmids established in this study are listed in Supplementary Table 4. The primers used for genotyping and cloning are listed in Supplementary Table 5. Synthetic sequences for plasmid construction are also listed in Supplementary Table 5.

All plasmids were assembled via the GreenGate cloning method^46^. DNA sequences of *ProEXP7* and *ProRHD6* were cloned from *Arabidopsis* genomic DNA using the primers listed in Supplementary Table 5 and were subsequently ligated to pGGA000 to form the promoter entry modules pGG-A-*ProEXP7*-B and pGG-A-*ProRHD6*-B, respectively. These two modules and the promoter entry module pGG-A-*ProUBQ10*+Ω-B^47^ were further assembled with the GreenGate modules pGGB003 (ref. ^46^), pSW596-mTurquoise2 (ref. ^48^) (Addgene plasmid no. 115985), pGGD007 (ref. ^46^), pGGE009 (ref. ^46^), pGG-F-Allired-G^47^ and pGGZ003 (refs. ^46,47^) to form the plasmids *ProUBQ10*::*mTurquoise2-NLS*, *ProRHD6*::*mTurquoise2-NLS* and *ProEXP7*::*mTurquoise2-NLS*, respectively.

For constructing plasmids for CRISPR-TSKO, we used an adapted method from ref. ^29^. The DNA fragments of two *ProU6-26*-driven guide RNAs for knocking out ATG5 (*ProU6-26-gATG5-1*_*ProU6-26-gATG5-2*; Supplementary Table 5) were cloned and inserted into pGGF000 to form the entry module pGG-F-*gATG5*-G. This entry module, together with the GreenGate modules pGG-B-Linker-C, pGG-C-Cas9PTA*-D, pGG-D-P2A-GFP-NLS-E, pGG-E-G7T-F and pFASTR-A-G^29^, was assembled with either pGG-A-*ProEXP7*-B or pGG-A-*ProRHD6*-B to form the recombinant plasmids *ProEXP7*::*Cas9-GFP*_*gATG5* and *ProRHD6*::*Cas9-GFP*_*gATG5*, respectively.

The coding sequence of *Arabidopsis* *ATG5* (Supplementary Table 3) was cloned into pTwist Amp High Copy to form the entry module pTwist-C-*ATG5*-D (Twist Bioscience). The GreenGate modules pGGB003 (ref. ^46^), pTwist-C-*ATG5*-D (Twist Bioscience), pGGD002 (ref. ^46^), pGGE009 (ref. ^46^), pGG-F-Allired-G^47^ and pGGZ003 (ref. ^46^) were assembled with either pGG-A-*ProEXP7*-B or pGG-A-*ProRHD6*-B to form the recombinant plasmids *ProEXP7*::*ATG5* and *ProRHD6*::*ATG5*, respectively.

All transgenic *Arabidopsis* lines were generated through the *Agrobacterium*-mediated floral-dip method^49^. *ProEXP7*::*Cas9-GFP*_*gATG5* and *ProRHD6*::*Cas9-GFP*_*gATG5* were transformed into *Arabidopsis* wild-type Col-0 plants, and the positive transformants were labelled as the lines TSKO-E and TSKO-R, respectively. *ProEXP7*::*ATG5* and *ProRHD6*::*ATG5* were transformed into *Arabidopsis* *atg5-1* mutant plants, and the positive transformants were labelled as the lines E-ATG5 and R-ATG5, respectively.

### Protein extraction for western blotting

*Arabidopsis* seeds were sterilized with 70% ethanol + 0.05% Tween 20 for 15 min and then with 100% ethanol for 15 min. Sterilized seeds were subsequently stored at 4 °C for 2 d for vernalization. Twenty to forty vernalized seeds were sown on 1/2 MS solid media (MS salt + Gamborg B5 vitamin mixture (Duchefa) supplemented with 0.5 g l^−1^ MES and 1% sucrose, pH 5.7) plates (+1% plant agar (Duchefa)) and were vertically grown as a bundle at 21 °C at 60% humidity under LEDs with a light intensity of 70 µM m^−2^s^−1^ and a 16 h light/8 h dark photoperiod for 7 d. Seven-day-old *Arabidopsis* seedlings were treated in either 1/2 MS ± 1 μM conA (CAS 80890-47-7; Santa Cruz) or 50 mM NaCl-containing 1/2 MS ± 1 μM conA liquid media for 12 h. The root parts of the seedlings were harvested in microcentrifugation tubes with different-sized glass beads (2.85–3.45 mm, 1.7–2.1 mm and 0.75–1.00 mm; Lactan GmbH) and flash-frozen in liquid nitrogen. The plants were ground with a mixer mill MM400 (4 × 30 s, 30 Hz; Retsch).

### Western blotting

All procedures of *Arabidopsis* protein extraction, protein concentration measurement and western blotting were followed as previously described^47^. Images were captured via the ChemiDoc Touch Imaging System (Bio-Rad).

### Plant phenotypic assays

*Arabidopsis* seeds were sterilized with 70% ethanol + 0.05% Tween 20 for 15 min and then with 100% ethanol for 15 min. Sterilized seeds were subsequently stored at 4 °C for 2 d for vernalization. Vernalized seeds were sown on 1/2 MS solid media (MS salt + Gamborg B5 vitamin mixture (Duchefa) supplemented with 0.5 g l^−1^ MES and 1% sucrose, pH 5.7) plates (+1% plant agar (Duchefa)) and were vertically grown at 21 °C at 60% humidity under LEDs with a light intensity of 70 µM m^−2^s^−1^ and a 16 h light/8 h dark photoperiod for 6 d. Six-day-old seedlings were subsequently transferred to 1/2 MS media plates (+1% plant agar (Duchefa)) containing 150 mM NaCl and vertically grown for 4 d. The seedlings were imaged at the day of transfer (d0) and at the fourth day after transfer (d4). The proportion of non-etiolated leaves to total leaves (including the cotyledons) of *Arabidopsis* seedlings at d4, the elongation length of the primary root and the newly emerged lateral roots at d4 compared to d0 were calculated for statistical analysis. The root length was measured via Fiji (v.1.54p, Fiji).

### Preparation of *A.thaliana* samples for confocal microscopy

*Arabidopsis* seeds were sterilized with 70% ethanol + 0.05% Tween 20 for 15 min and then with 100% ethanol for 15 min. Sterilized seeds were subsequently stored at 4 °C for 2 d for vernalization. Vernalized seeds were spread on 1/2 MS media plates (+1% plant agar (Duchefa)) and vertically grown at 21 °C at 60% humidity under LEDs with a light intensity of 70 µM m^−2^s^−1^ and a 16 h light/8 h dark photoperiod for 5 d. Five-day-old seedlings were placed in 1/2 MS media and treated with salt or chemicals as indicated in each experiment before confocal imaging. CoroNa Green AM (Invitrogen C36676), CM-H_2_DCFDA (Invitrogen C6827) and conA (CAS 80890-47-7; Santa Cruz) was dissolved in DMSO and added to the desired stock concentration (2 mM for CoroNa Green AM, 10 mM for CM-H_2_DCFDA and 2 mM for conA). For nitrogen starvation, 1/2 MS media was replaced with nitrogen-deficient 1/2 MS media (MS salt without nitrogen (Caisson Labs) + Gamborg B5 vitamin mixture (Duchefa) supplemented with 0.5 g l^−1^ MES and 1% sucrose, pH 5.7).

For confocal microscopy, 5-d-old *Arabidopsis* seedlings were placed on a microscope slide with either water or water with propidium iodide (0.002 mg ml^−1^ for ZEISS LSM800 and 0.01 mg ml^−1^ for Stellaris 8 Confocal) and covered with a coverslip.

### Confocal microscopy

All images except those in Fig. 6 and Extended Data Fig. 7 were acquired using an upright point laser scanning confocal microscope ZEISS LSM800 Axio Imager.Z2 (Carl Zeiss) equipped with high-sensitive GaAsP detectors (Gallium Arsenide), a Plan-Apochromat ×20 objective lens (numerical aperture 0.8, dry), a LD C-Apochromat ×40 objective lens (numerical aperture 1.1, water; only for cotyledon epidermis imaging in Extended Data Fig. 6e) and ZEN software (blue edition, Carl Zeiss). For Fig. 6 and Extended Data Fig. 7, images were acquired using a Leica Stellaris 8 confocal microscope equipped with an integrated white light laser, the Power HyD detector family, a HC PL APO CS2 ×20 objective lens (numerical aperture 0.75, dry) and LAS X software. The fluorescence of GFP, mTurquoise2, CoroNa Green AM and CM-H_2_DCFDA was excited at 488 nm. The fluorescence of propidium iodide and mCherry was excited at 561 nm. For *Z*-stack imaging, the interval between layers was set as 1 μm. Confocal images were processed with Fiji (v.1.54p, Fiji).

For autophagic flux quantification, only the round GFP–ATG8-labelled puncta with a size between 0.10 and 4.00 μm^2^ were counted as autophagosomes or autophagic bodies inside the vacuole, while the GFP–ATG8-labelled spindle-shaped ER bodies in the *wer* *myb23* mutant line were not counted.

### Transmission electron microscopy

High-pressure freezing, freeze-substitution, low-temperature embedding, ultramicrotomy and transmission electron microscopy imaging were carried out according to a protocol described previously^50^. Briefly, 5-d-old *Arabidopsis* seedlings were incubated in 50 mM NaCl-containing 1/2 MS liquid media for 45 min for autophagy induction. Subsequently, the roots of the seedlings were cryofixed with an HPM100 high-pressure freezer (Leica Microsystems) and incubated in freeze-substitution medium (anhydrous acetone with 0.25% glutaraldehyde and 0.1% uranyl acetate) for two days at −80 °C. After the incubation temperature was raised to −50 °C (1 °C h^−1^), the freeze-substitution medium was washed with anhydrous acetone, and the samples were embedded in HM20 resin (Ted Pella) over two days at −50 °C. The resin was cured by UV illumination (24 h) at −50 °C. An AFS2 machine (Leica Microsystems) was used for freeze-substitution, resin embedding and polymerization.

For immunogold labelling of the *Arabidopsis* mutant line *wer* *myb23* expressing *GFP-ATG8A*, primary antibody anti-GFP (Rabbit Polyclonal; Rockland, 600-401-215) was diluted 1:40, and then the primary antibody was visualized with anti-rabbit IgG (10 nm; SKU.25109, Electron Microscopy Sciences) secondary antibody conjugated with gold particles. Preparation of thin sections (100 nm) and immunogold labelling were performed as previously described^50,51^. After post-staining with uranyl acetate and Reynolds lead citrate solutions, the sections were examined with a transmission electron microscope (Hitachi H-7650) operated at 80 kV.

### Statistical analyses

All quantification analyses and statistical tests were performed with GraphPad Prism 10 software (v.10.5.0, GraphPad Software, www.graphpad.com). For comparing the significance of differences between two experimental groups, paired and two-tailed Student’s *t*-tests were performed as indicated in each experiment. For comparing the significance of differences between multiple experimental groups, paired repeated-measures one-way or two-way ANOVA and Fisher’s LSD tests were performed as indicated in each experiment.

### Single-cell RNA-seq analysis

Single-cell RNA-seq analysis was performed using R (v.4.4.1)^52^ and RStudio (v.2024.12.0)^53^. Three published single-cell RNA-seq datasets of *A.* *thaliana* were chosen as reference datasets 1 (ref. ^40^), 2 (ref. ^41^) and 3 (ref. ^42^) for screening the genes that are specifically highly expressed in trichoblasts or atrichoblasts. Cell-type annotations provided in each dataset were used to extract trichoblast and atrichoblast cell populations. Marker genes specific to trichoblasts or atrichoblasts were identified using the R package Seurat (v.4.2.1)^54^ with the function FindAllMarkers. Genes with both adjusted *P* < 0.05 and log_2_ fold change (log_2_FC) > 0.36 were screened out as specifically highly expressed genes in either trichoblasts or atrichoblasts. The resulting gene lists for trichoblasts and atrichoblasts were compared across all three datasets. Venn diagrams were generated using the R package VennDiagram (v.1.7.3)^55^.

For the genes screened out in all three datasets, the log_2_FC values from each dataset were extracted and standardized (*Z*-score normalization) to emphasize relative expression patterns across datasets. The *Z*-score was calculated as the difference value between the log_2_FC of each reference and the mean log_2_FC of three references divided by the standard deviation. Heat maps were generated using the online platform https://hiplot.cn.

The Gene Ontology (GO) enrichment analysis was performed using the clusterProfiler package (v.4.12.6)^56^. The enrichGO function was used to identify significantly enriched GO terms, and multiple hypothesis testing was corrected using the Benjamini–Hochberg method (pAdjustMethod, BH). GO terms with an adjusted *P* < 0.05 (pvalueCutoff, 0.05) were considered significantly enriched.

### Reporting summary

Further information on research design is available in the Nature Portfolio Reporting Summary linked to this article.

## Supplementary information

Supplementary Information Supplementary Fig. 1 and source data for Supplementary Fig. 1.

Reporting Summary

Supplementary Table 1 Genes that are specifically more highly expressed in trichoblasts than in atrichoblasts.

Supplementary Table 2 Genes that are specifically more highly expressed in atrichoblasts than in trichoblasts.

Supplementary Table 3 *Arabidopsis thaliana* lines used in this study.

Supplementary Table 4 List of plasmids used in this study.

Supplementary Table 5 Primers and synthetic sequences used in this study for genotyping and cloning.

## Source data

Source Data Extended Data Fig. 4 Unprocessed western blots.

## Data Availability

All the data presented in this manuscript are available via Zenodo at 10.5281/zenodo.18243590 (ref. ^57^) and 10.5281/zenodo.18243608 (ref. ^58^). Source data are provided with this paper.
