# Supplementary material for: Cell-type-specific autophagy in root-hair-forming cells is essential for salt stress tolerance in Arabidopsis thaliana
Source: Nat Plants. 2026 May 6;12(5):1008–21. doi: 10.1038/s41477-026-02285-w (PMC13197226; doi:10.1038/s41477-026-02285-w)
Supplement: Supplementary file 1 — Supplementary Fig. 1 and source data for Supplementary Fig. 1. [file 41477_2026_2285_MOESM1_ESM.pdf]

nature plants

Supplementary information

https://doi.org/10.1038/s41477-026-02285-w

Cell-type-specific autophagy in root-hair-forming cells is essential for salt stress tolerance in *Arabidopsis thaliana*

In the format provided by the authors and unedited

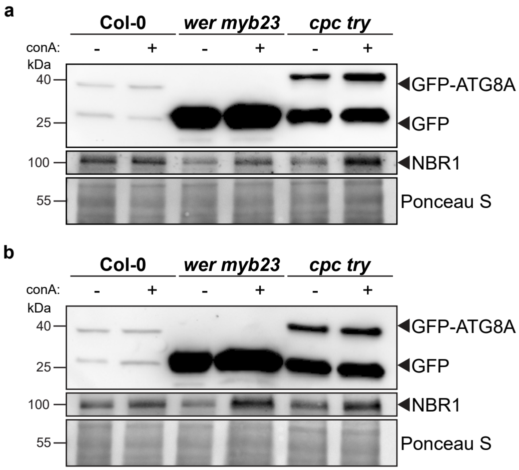

a
Col-0
wer myb23
cpc try
conA:
-
+
-
+
-
+
kDa
40
25
100
55
GFP-ATG8A
GFP
NBR1
Ponceau S
b
Col-0
wer myb23
cpc try
conA:
-
+
-
+
-
+
kDa
40
25
100
55
GFP-ATG8A
GFP
NBR1
Ponceau S

Supplementary Fig. 1 | The second replicate of Extended Data Fig. 4d and 4e.

a-b. Western blots showing GFP-ATG8A cleavage level and endogenous NBR1 level in wildtype Col-0, *wer myb23* and *cpc try* mutants under control (**a**) or NaCl (**b**) treatment. 7 days old Arabidopsis seedlings were treated in either 1/2 MS ± 1 μM conA (**a**) or 50 mM NaCl-containing 1/2 MS ± 1 μM conA (**b**) liquid media for 12 h. Ten μg of total protein extract of the root sections was loaded and immunoblotted with anti-GFP and anti-NBR1 antibodies.

Source data for Supplementary Fig. 1

1a. anti-GFP

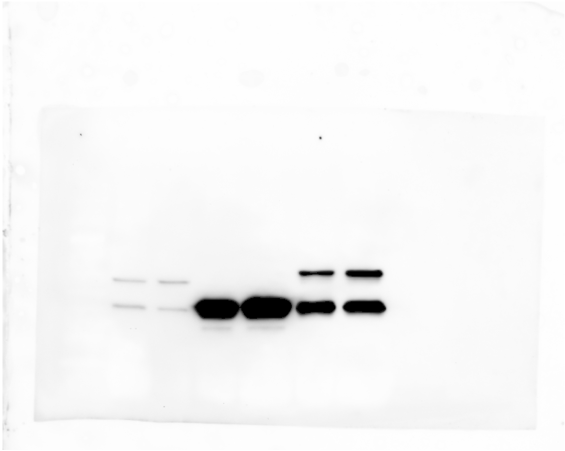

1b. anti-GFP

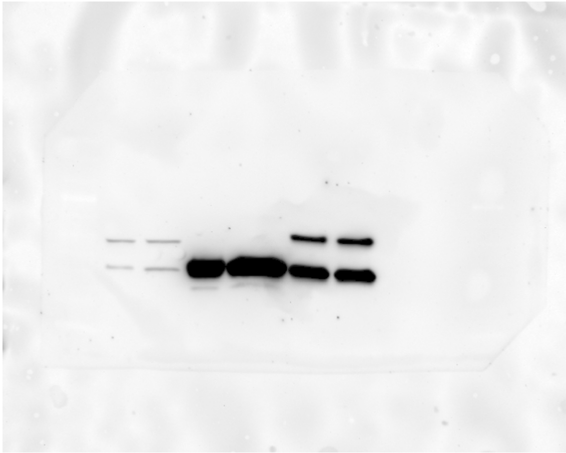

1a. anti-NBR1

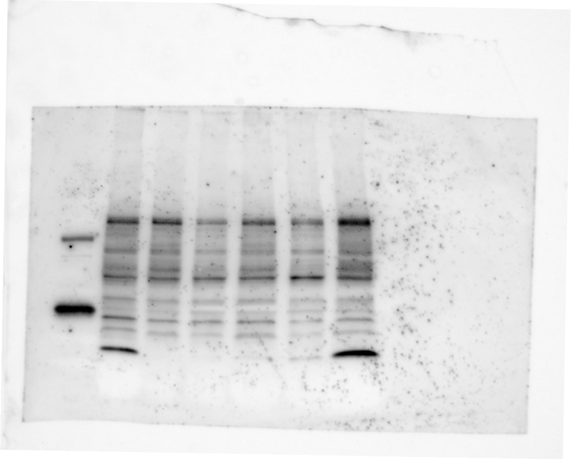

1b. anti-NBR1

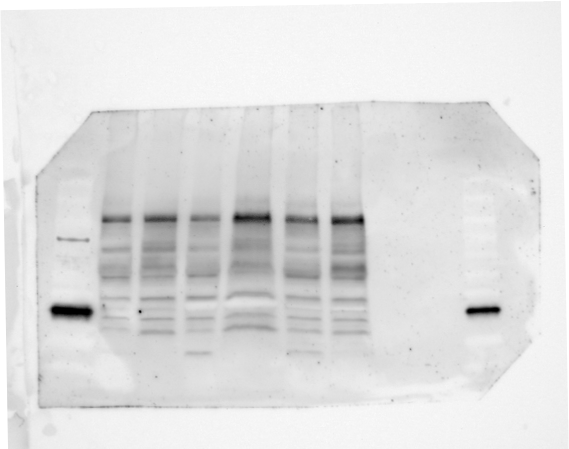

1a. Ponceau S

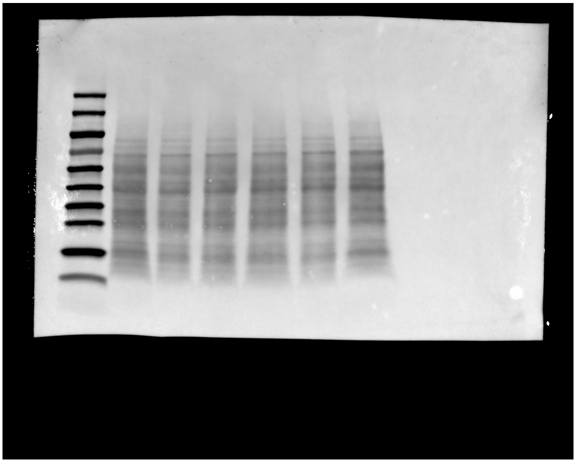

1b. Ponceau S

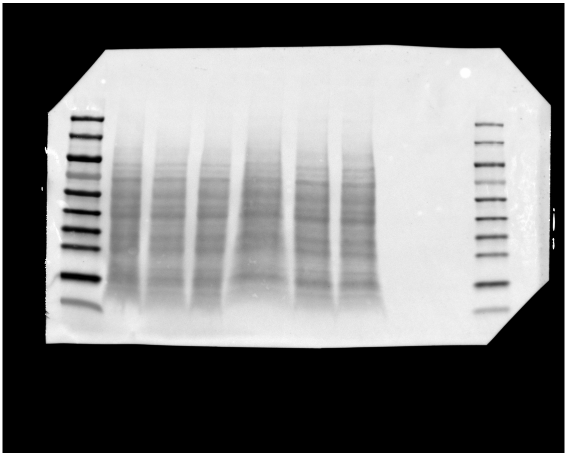

Supplementary references

1. Thompson, A. R., Doelling, J. H., Suttangkakul, A. & Vierstra, R. D. Autophagic nutrient recycling in Arabidopsis directed by the ATG8 and ATG12 conjugation pathways. *Plant Physiol* 138, 2097–2110 (2005).
2. Del Chiaro, A. *et al.* An A. thaliana mutant lacking all nine ATG8 isoforms provides genetic evidence for functional specialization of ATG8 in plants. *J Cell Sci* 138, 1–12 (2025).
3. Julian, J. *et al.* ATG8ylation of vacuolar membrane protects plants against cell wall damage. *Nat Plants* 11, 323–339 (2025).
4. Menand, B. *et al.* An ancient mechanism controls the development of cells with a rooting function in land plants. *Science (1979)* 316, 1477–1480 (2007).
5. Schellmann, S. *et al.* TRIPTYCHON and CAPRICE mediate lateral inhibition during trichome and root hair patterning in Arabidopsis. *EMBO J* (2002).
6. Lee, M. M. & Schiefelbein, J. WEREWOLF, a MYB-related protein in Arabidopsis, is a position-dependent regulator of epidermal cell patterning. *Cell* 99, 473–483 (1999).
7. Lee, M. M. & Schiefelbein, J. Developmentally distinct MYB genes encode functionally equivalent proteins in Arabidopsis. *Development* 128, 1539–1546 (2001).
8. Munch, D. *et al.* Retromer contributes to immunity-associated cell death in Arabidopsis. *Plant Cell* 27, 463–479 (2015).
9. Stephani, M. *et al.* A cross-kingdom conserved ER-phagy receptor maintains endoplasmic reticulum homeostasis during stress. *Elife* 9, e58396 (2020).
10. Hu, S., Ye, H., Cui, Y. & Jiang, L. AtSec62 is critical for plant development and is involved in ER-phagy in Arabidopsis thaliana. *J Integr Plant Biol* 62, 181–200 (2020).
