# Supplementary material for: Cell-type-specific autophagy in root-hair-forming cells is essential for salt stress tolerance in Arabidopsis thaliana
Source: Nat Plants. 2026 May 6;12(5):1008–21. doi: 10.1038/s41477-026-02285-w (PMC13197226; doi:10.1038/s41477-026-02285-w)
Supplement: Supplementary file 2 — Reporting Summary [file 41477_2026_2285_MOESM2_ESM.pdf]

nature portfolio

Corresponding author(s): Jierui Zhao, Yasin Dagdas

Last updated by author(s): Mar 5, 2026

Reporting Summary

Nature Portfolio wishes to improve the reproducibility of the work that we publish. This form provides structure for consistency and transparency in reporting. For further information on Nature Portfolio policies, see our Editorial Policies and the Editorial Policy Checklist.

Statistics

For all statistical analyses, confirm that the following items are present in the figure legend, table legend, main text, or Methods section.

n/a	Confirmed	
☐	☒	The exact sample size (*n*) for each experimental group/condition, given as a discrete number and unit of measurement
☐	☒	A statement on whether measurements were taken from distinct samples or whether the same sample was measured repeatedly
☐	☒	The statistical test(s) used AND whether they are one- or two-sided<br>*Only common tests should be described solely by name; describe more complex techniques in the Methods section.*
☒	☐	A description of all covariates tested
☐	☒	A description of any assumptions or corrections, such as tests of normality and adjustment for multiple comparisons
☐	☒	A full description of the statistical parameters including central tendency (e.g. means) or other basic estimates (e.g. regression coefficient) AND variation (e.g. standard deviation) or associated estimates of uncertainty (e.g. confidence intervals)
☐	☒	For null hypothesis testing, the test statistic (e.g. *F*, *t*, *r*) with confidence intervals, effect sizes, degrees of freedom and *P* value noted<br>*Give P values as exact values whenever suitable.*
☒	☐	For Bayesian analysis, information on the choice of priors and Markov chain Monte Carlo settings
☒	☐	For hierarchical and complex designs, identification of the appropriate level for tests and full reporting of outcomes
☒	☐	Estimates of effect sizes (e.g. Cohen's *d*, Pearson's *r*), indicating how they were calculated

Our web collection on statistics for biologists contains articles on many of the points above.

Software and code

Policy information about availability of computer code

Data collection	ZEISS ZEN Blue 3.8, Leica LAS X
Data analysis	Fiji/ImageJ v1.54p, GraphPad Prism 10.5.0, R (version 4.4.1), RStudio (version 2024.12.0; the R package Seurat version 4.2.1; the R package ”VennDiagram” version 1.7.3; the clusterProfiler package version 4.12.6)

For manuscripts utilizing custom algorithms or software that are central to the research but not yet described in published literature, software must be made available to editors and reviewers. We strongly encourage code deposition in a community repository (e.g. GitHub). See the Nature Portfolio guidelines for submitting code & software for further information.

Data

Policy information about availability of data

All manuscripts must include a data availability statement. This statement should provide the following information, where applicable:

- Accession codes, unique identifiers, or web links for publicly available datasets
- A description of any restrictions on data availability
- For clinical datasets or third party data, please ensure that the statement adheres to our policy

All the source data associated with the data presented in this manuscript is available at Zenodo. DOI: 10.5281/zenodo.18243590 and DOI: 10.5281/zenodo.18243608

Research involving human participants, their data, or biological material

Policy information about studies with human participants or human data. See also policy information about sex, gender (identity/presentation), and sexual orientation and race, ethnicity and racism.

Reporting on sex and gender	n/a
Reporting on race, ethnicity, or other socially relevant groupings	n/a
Population characteristics	n/a
Recruitment	n/a
Ethics oversight	n/a

Note that full information on the approval of the study protocol must also be provided in the manuscript.

Field-specific reporting

Please select the one below that is the best fit for your research. If you are not sure, read the appropriate sections before making your selection.

☒ Life sciences ☐ Behavioural & social sciences ☐ Ecological, evolutionary & environmental sciences

For a reference copy of the document with all sections, see nature.com/documents/nr-reporting-summary-flat.pdf

Life sciences study design

All studies must disclose on these points even when the disclosure is negative.

Sample size	Sample sizes were selected based on experimental logistics in all experiments mentioned in the manuscript. The consistency of results within and between replicates was closely monitored, and the variability observed was minimal. This confirmed that our chosen sample size was sufficient to draw reliable conclusions for the scope of this study.
Data exclusions	No data were excluded.
Replication	All experiments in this study were measured at least 2 times with comparable results and no unsuccessful replication.
Randomization	No experimental group was used in this study.
Blinding	Blinding was not performed in this study because the experimental design inherently minimized the risk of bias. All images and raw data from all samples were acquired and stored systematically, enabling transparent re-analysis at any time. Additionally, the nature of the experiments - such as the use of quantifiable image analysis (e.g., puncta counting, intensity measurements) - limited the potential for subjective interpretation. The consistency of results within and between replicates further supports the reliability of our conclusions. If necessary, stored data could be scored independently by additional researchers to confirm findings.

Reporting for specific materials, systems and methods

We require information from authors about some types of materials, experimental systems and methods used in many studies. Here, indicate whether each material, system or method listed is relevant to your study. If you are not sure if a list item applies to your research, read the appropriate section before selecting a response.

Materials & experimental systems

n/a	Involved in the study	
☐	☒	Antibodies
☒	☐	Eukaryotic cell lines
☒	☐	Palaeontology and archaeology
☒	☐	Animals and other organisms
☒	☐	Clinical data
☒	☐	Dual use research of concern
☐	☒	Plants

Methods

n/a	Involved in the study	
☒	☐	ChIP-seq
☒	☐	Flow cytometry
☒	☐	MRI-based neuroimaging

Antibodies

Antibodies used	1. Mouse monoclonal anti-GFP (diluted by 1:3000 v/v, 11814460001; Roche) was used for western blotting;<br>2. Rabbit polyclonal anti-NBR1 (diluted by 1:2000 v/v, AS14 2805; Agrisera) was used for western-blotting;<br>3. Mouse monoclonal antibody for western blotting were detected with goat anti-mouse IgG HRP-linked antibody (diluted by 1:5000 v/v, 61-6520, Invitrogen) second antibody;<br>4. Rabbit monoclonal antibody for western blotting were detected with a goat anti-rabbit IgG HRP-linked antibody (diluted by 1:5000 v/v, 65-6120, Invitrogen) second antibody;<br>5. The primary antibody anti-GFP (diluted by 1:40 v/v, Rabbit Polyclonal; Rockland, 600-401-215) was used for TEM;<br>6. The second antibody anti-rabbit IgG (10nm; SKU.25109, Electron 447 Microscopy Sciences) conjugated with gold particles was used for TEM.
Validation	All the antibodies used in this study were commercial antibodies to standard epitope tags, validated by manufacturers. Reference papers of primary antibodies provided by manufacturers:<br>1. Mouse monoclonal anti-GFP:<br>Crameri A, et al., Nature Biotechnology, 14, 315-319 (1996) Defective ribosome assembly in Shwachman-Diamond syndrome.<br>2. Rabbit polyclonal anti-NBR1:<br>Guan et al. (2025).Arabidopsis phospholipase Dζ2 facilitates vacuolar acidification and autophagy under phosphorus starvation by interacting with VATD. Cell Rep. 2025 Jul 15;44(7):116024. doi: 10.1016/j.celrep.2025.116024.<br>3. Rabbit polyclonal anti-GFP:<br>Fay A et al. (2025). A split ALFA tag-nanobody system for protein localization and proximity proteomics in mycobacteria. mBio.

Dual use research of concern

Policy information about dual use research of concern

Hazards

Could the accidental, deliberate or reckless misuse of agents or technologies generated in the work, or the application of information presented in the manuscript, pose a threat to:

No	Yes	
☒	☐	Public health
☒	☐	National security
☒	☐	Crops and/or livestock
☒	☐	Ecosystems
☒	☐	Any other significant area

Experiments of concern

Does the work involve any of these experiments of concern:

No	Yes	
☒	☐	Demonstrate how to render a vaccine ineffective
☒	☐	Confer resistance to therapeutically useful antibiotics or antiviral agents
☒	☐	Enhance the virulence of a pathogen or render a nonpathogen virulent
☒	☐	Increase transmissibility of a pathogen
☒	☐	Alter the host range of a pathogen
☒	☐	Enable evasion of diagnostic/detection modalities
☒	☐	Enable the weaponization of a biological agent or toxin
☒	☐	Any other potentially harmful combination of experiments and agents

Plants

Seed stocks

No plant specimen was collected from the field.
All seed stocks are described in Supplementary Table 3.

Novel plant genotypes

All transgenic Arabidopsis lines were generated through the Agrobacterium-mediated floral-dip method (Clough and Bent, 1998). All novel plant genotypes are decribed in Supplementary Table 3. For R-ATG5 and E-ATG5, only 1 positive transformant is examined and used for further experiments as the transgenic efficieny to Arabidopsis atg5 is very low. For all other lines, at least 3 independent positive T1 transformants were analyzed and went for reproduction to T2 generation. ProPYK10:PYK10-TagRFP x ProUBQ10:GFP-ATG8A in Col-0 and wer myb23, ProUBQ10:GFP-ATG8A x ProUBQ10:DDRGK1-mCherry in Col-0 and wer myb 23, ProSOS1:SOS1-mCitrine x ProUBQ10:mCherry-ATG8E, ProUBQ10:NHX1-GFP x ProUBQ10:mCherry-ATG8E, ProUBQ10:mTurquoise2-NLS, ProEXP7:mTurquoise2-NLS and ProRHD6:mTurquoise2-NLS were T2 generation and were at heterozygous stage. All the other transgenic and crossed lines used for all experiments in this study were at least T3 generation and were homozygous.

Authentication

All primers used for genotyping the gl2-8, wer-1 myb23-1 are described in Table 3. For gl2-8 and myb23, the authentication procedure is described as shown in http://signal.salk.edu/tdnaprimers.2.html#opennewwindow. For wer-1 genotyping, the PCR cloning product was subsequently digested with MboI to generate 2 fragments in the wer1 mutant while the fragments cloned from wild-type genome will not be cut into 2 parts. All transfenic Arabidopsis lines were authenticated through fluorescent protein imaging under confocal microscopy.
