# Supplementary figures and images for: Cell-type-specific autophagy in root-hair-forming cells is essential for salt stress tolerance in Arabidopsis thaliana
Source: Nat Plants. 2026 May 6;12(5):1008–21. doi: 10.1038/s41477-026-02285-w (PMC13197226; doi:10.1038/s41477-026-02285-w)

4d. anti-GFP

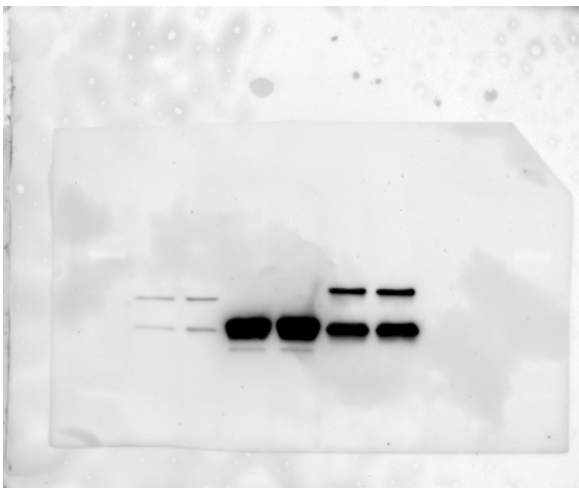

4e. anti-GFP

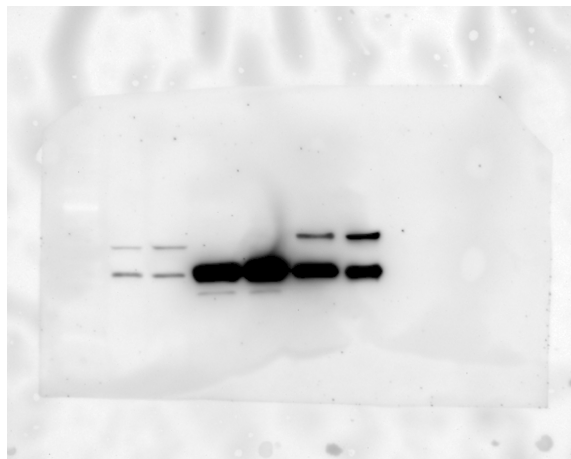

4d. anti-NBR1

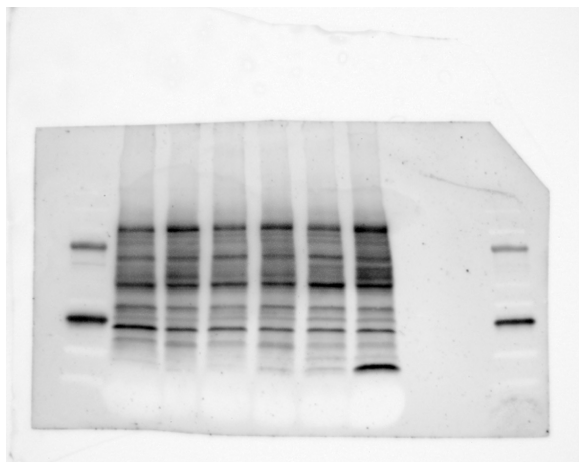

4e. anti-NBR1

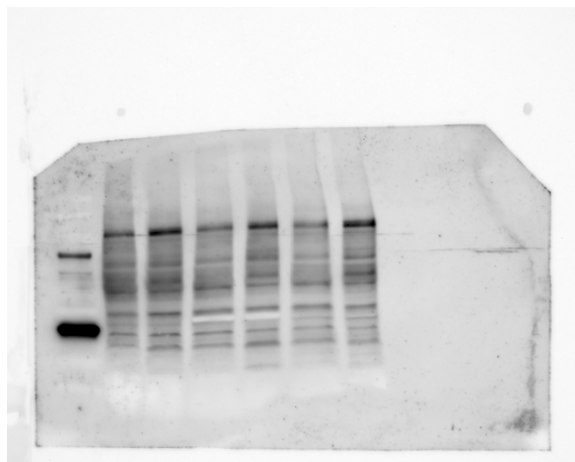

4d. Ponceau S

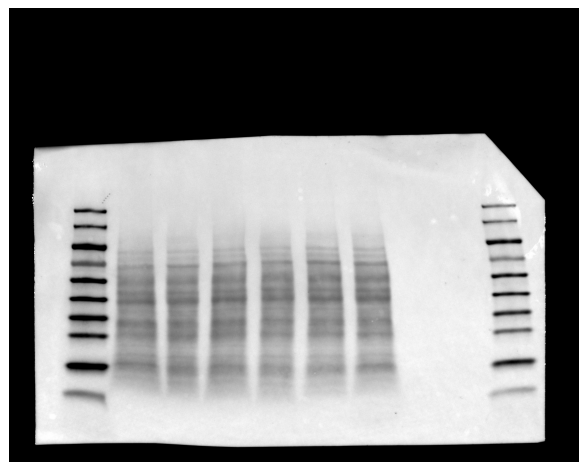

4e. Ponceau S

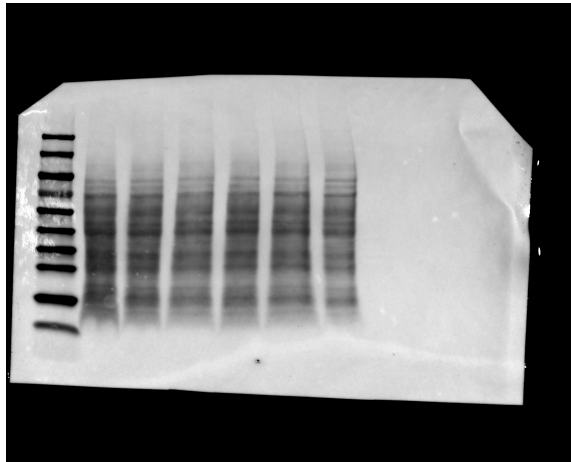

Supplement: Supplementary file 8 — Unprocessed western blots. [file 41477_2026_2285_MOESM8_ESM.pdf]
